# Discovery of two-dimensional binary nanoparticle superlattices using global Monte Carlo optimization

**DOI:** 10.1038/s41467-022-35690-8

**Published:** 2022-12-29

**Authors:** Yilong Zhou, Gaurav Arya

**Affiliations:** 1 Department of Mechanical Engineering and Materials Science, Duke University, Durham, NC 27708 USA; 2 Present Address: Materials Science Division, Lawrence Livermore National Laboratory, Livermore, CA 94550 USA

**Keywords:** Nanoparticles, Computational methods, Two-dimensional materials

## Abstract

Binary nanoparticle (NP) superlattices exhibit distinct collective plasmonic, magnetic, optical, and electronic properties. Here, we computationally demonstrate how fluid-fluid interfaces could be used to self-assemble binary systems of NPs into 2D superlattices when the NP species exhibit different miscibility with the fluids forming the interface. We develop a basin-hopping Monte Carlo (BHMC) algorithm tailored for interface-trapped structures to rapidly determine the ground-state configuration of NPs, allowing us to explore the repertoire of binary NP architectures formed at the interface. By varying the NP size ratio, interparticle interaction strength, and difference in NP miscibility with the two fluids, we demonstrate the assembly of an array of exquisite 2D periodic architectures, including AB-, AB_2_-, and AB_3_-type monolayer superlattices as well as AB-, AB_2_-, A_3_B_5_-, and A_4_B_6_-type bilayer superlattices. Our results suggest that the interfacial assembly approach could be a versatile platform for fabricating 2D colloidal superlattices with tunable structure and properties.

## Abstract (web summary)

Binary nanoparticle superlattices exhibit different collective optical, magnetic, and electronic properties. Here, the authors develop an efficient global optimization algorithm for the discovery of periodic 2D architectures forming at fluid interfaces.

## Introduction

NP superlattices have attracted considerable scientific interest because of their potential applications in plasmonic^1–3^, optoelectronic^4^, photonic^5^, and phononic^6,7^ devices as well as in catalysis^8,9^ and energy conversion^10,11^. Compared to single-component NP superlattices, binary NP superlattices (BNSLs)—composed of two types of NPs differing in size or composition—possess significantly more structural and chemical diversity. As a result, BNSLs can exhibit collective properties distinct from those of individual NPs, disordered NP structures, and single-component NP superlattices^12–14^. For instance, BNSLs assembled from plasmonic and non-plasmonic NPs exhibit a collective plasmonic response that can be tuned across the entire visible spectrum by varying NP size, composition and lattice symmetry^15^. Among them, 2D BNSLs are especially attractive because they could also be used as templates in colloidal lithography for fabricating nanostructured materials^16^ and applications such as electroluminescence prefer the NPs to be assembled into monolayers rather than 3D structures^17,18^.

Interfaces formed between mutually immiscible fluids provide a powerful platform for assembling NPs into 2D higher-order structures^19^. However, spherical NPs tend to form hexagonally packed monolayers at fluid-fluid interfaces to maximize interparticle contacts^20^. Recently, we proposed a strategy for assembling more unusual structures from spherical NPs trapped at interfaces^21^. The approach takes advantage of the phenomenon where particles get increasingly displaced from the interfacial plane with increasing difference in the surface energy of NPs with the two fluids. Such differences could be engineered by grafting NP surfaces with ligands that have different miscibility with the two fluids. By co-assembling multiple species of grafted NPs, where species get trapped in distinct planes parallel to the interfacial plane based on how differently their grafts interact with the two fluids, we demonstrated via coarse-grained molecular dynamics (CGMD) simulations that such NPs could be assembled into distinct clusters, strings, and sheets. Among layered structures relevant to 2D BNSLs, we successfully assembled a ridged monolayer and a square-packed interdigitated bilayer. While these examples illustrate the potential of our interfacial assembly approach for fabricating 2D BSNLs, only a handful of assembly scenarios could be investigated due to the high computational cost of MD simulations, especially for superlattices with large, complex unit cells. Therefore, the full potential of this approach for self-assembling 2D BNSLs could not be explored. Furthermore, there is no guarantee in MD simulations that the final, assembled NP configuration represents the globally stable state, as configurations often get trapped in metastable states. In fact, the number of such locally stable states increases exponentially with system size, and so do the number of transition states^22,23^. The task of obtaining globally stable NP structures is thus a global optimization problem. Many optimization algorithms have been developed for molecular and crystal structure prediction^24–30^, but none are applicable to studying interfacial assembly of NPs. Similarly, although some computational efforts have been devoted to exploration of NP superlattices^31–37^, none of them have focused on 2D BNSLs formed at interfaces.

**Fig. 1:**
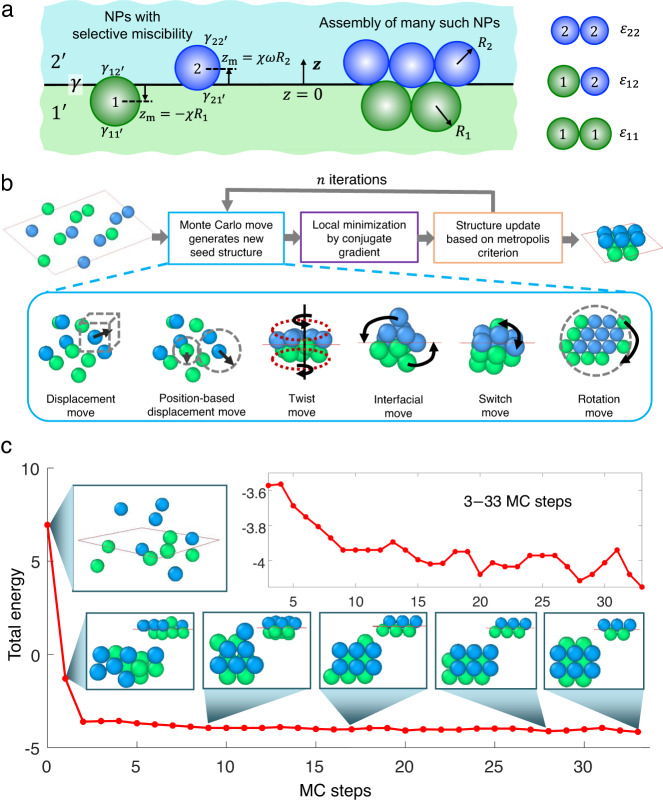
Global optimization approach for exploring NP structures formed at fluid interfaces. **a** Schematic of the model system comprised of two species of surface-functionalized NPs assembling in a fluid-fluid bilayer and the parameters used for describing its free energy. **b** Simplified flow chart of our tailored BHMC algorithm highlighting the MC moves we implemented to improve performance. **c** Optimization outcome for a binary 6:6 system of NPs ($\varphi=1$, $\epsilon=0.08$, $\chi {{{{{\boldsymbol{=}}}}}}0.6$, $\omega=1$) that yields a square-packed bilayer as the global minimum. The total energy (normalized by $\pi {R}_{1}^{2}\gamma$) vs. MC steps and the various structures obtained during the optimization process are shown. The large initial drop in energy is attributed to a transition from a loosely dispersed state of NPs to a relatively dense planar structure. The structure corrects itself in subsequent steps of optimization, transitioning through increasingly less-defective square-packed bilayer structures. At the penultimate step, the algorithm obtains a square-packed bilayer with parallel-aligned NP layers, before yielding the globally stable square-packed bilayer with perpendicularly aligned layers.

In this work, we develop an efficient global optimization approach tailored for obtaining the ground-state structure of interface-trapped NP assemblies and explore the full range of structural phases that binary systems of NPs form at fluid-fluid interfaces (Fig. 1). By examining a range of NP size ratios, interparticle interaction strengths, and surface tension differences of NPs with the liquid bilayer, we uncover many interesting monolayer, bilayer, and globular structures. By analyzing large-scale configurations of the obtained monolayer and bilayer clusters, many unusual 2D BNSLs structures are revealed. The optimization approach designed here, the assembly strategy explored here, the understandings gained here, and the structures discovered here, all represent major advancements in the synthesis and discovery of 2D materials.

## Results

### Model development and structure optimization

The system of interest comprises of two species of surface-functionalized spherical NPs (labelled 1 and 2) trapped at a planar interface (located at position $z=0$) formed between two mutually immiscible fluids (denoted by 1’ for fluid layer at $z\, < \,0$ and 2’ for layer at $z\, > \,0$) in which the two types of NPs interact favorably with opposite layers of the fluid bilayer, that is, NP 1 with fluid 1’ and NP 2 with fluid 2’ (Fig. 1a). To keep computational costs manageable, we used a simplified model of the system that captures the underlying physics of NP assembly, namely, the competition between interparticle interactions and interfacial interactions of the two NPs with the two fluid layers.

As detailed in Methods, our model treats the two types of NPs as spheres of radii ${R}_{1}$ and ${R}_{2}$ that interact with each other via short-ranged potentials ${U}_{{ij}}$ (${ij}$ = 11, 22, and 12 denotes interactions between like and unlike pairs of NPs) whose interaction strengths ${\varepsilon }_{{ij}}$ were assumed to be proportional to NP radii, consistent with the size scaling of van der Waals interactions between spherical colloids (Supplementary Fig. 1)^38^. Throughout this work, we fix ${R}_{1}=3\sigma$ ($\sigma$ represents an intrinsic length scale of the system) and vary the radius ${R}_{2}$ of the other NP type. To describe the interaction of NPs with the fluid bilayer, we used an analytical model we recently developed^21^ for the position ($z$)-dependent free energy $\triangle {F}_{i}$ of a surface-functionalized NP. The model accounts for the surface tension $\gamma$ between the fluids, which attempts to trap the NPs symmetrically about the interfacial plane so that they occlude the largest possible interfacial area, and difference in the surface energy $\triangle {\gamma }_{i}$ of the NPs with the two fluids, which attempts to expel NPs from their less favored fluid layer (Supplementary Fig. 2). The interplay between these two effects determines the equilibrium position ${z}_{{{{{{\rm{m}}}}}}}$ of the NPs (in the absence of interparticle interactions):

1 $${z}_{{{{{{\rm{m}}}}}}}=\pm \frac{\triangle {\gamma }_{i}}{\gamma }{R}_{i}$$

2 $$\triangle {F}_{i}\left({z}_{{{{{{\rm{m}}}}}}}\right)=-\pi {R}_{i}^{2}\gamma {\left(1-\frac{\triangle {\gamma }_{i}}{\gamma }\right)}^{2}$$

where the sign in Eq. (1) is “$-$” for NP 1 and “$+$” for NP 2, and $\triangle {F}_{i}\left({z}_{{{{{{\rm{m}}}}}}}\right)$ is the interfacial free energy of the NP at this position. The total energy of a binary system of $N$ NPs (${N}_{1}$ of type 1 and ${N}_{2}$ of type 2, so $N={N}_{1}+{N}_{2}$) is then represented by the sum of $N(N-1)/2$ pairwise interactions between the NPs and $N$ interfacial energy contributions from individual particles.

Dimensional analysis over the energy expressions leads to four dimensionless parameters that entirely describe the assembly thermodynamics of the NPs:

3 $$\varphi=\frac{{R}_{2}}{{R}_{1}}$$

4 $$\epsilon=\frac{{\varepsilon }_{11}}{\pi {R}_{1}^{2}\gamma }$$

5 $$\chi=\frac{\triangle {\gamma }_{1}}{\gamma }$$

6 $$\omega=\frac{\triangle {\gamma }_{2}}{\triangle {\gamma }_{1}}$$

where $\varphi$ is the size ratio of the two species of NPs, $\epsilon$ is the relative strength of interparticle interaction ${\varepsilon }_{11}$ to surface tension, $\chi$ is the ratio of the difference in the surface energy of NP 1 with the two fluids to the surface tension between the two fluids (this parameter also determines the equilibrium position of NPs as seen from Eq. (1)), and $\omega$ is the ratio of the differences in the surface energy of the two types of NPs with the two layers. Table 1 lists the parameter values whose combinations are explored in this study.

**Table 1:** Dimensionless parameter values explored in this work

Parameters	Values
${{{{{\boldsymbol{\varphi }}}}}}$	1/3, 1/2, 2/3, 5/6, 1
${{{{{\boldsymbol{\epsilon }}}}}}$	0.02, 0.04, 0.08, 0.16, 0.32, 0.64, 1.28, 2.56, 5.12
${{{{{\boldsymbol{\chi }}}}}}$	0, 0.2, 0.4, 0.6, 0.8, 1
${{{{{\boldsymbol{\omega }}}}}}$	0, 0.5, 1, 2

To locate the global minimum-energy (ground-state) configuration of NPs for each parameter combination, we used the BHMC global optimization algorithm^39–41^. However, the NP displacement “moves” implemented in standard BHMC algorithms are not particularly efficient for sampling NP configurations of the quasi-2D morphologies explored in this work^13,19,21^. To this end, we designed several new MC moves for improving the sampling efficiency of the BHMC algorithm (Fig. 1b). To demonstrate the efficiency of our tailored BHMC algorithm, we present in Fig. 1c the evolution in total energy with MC steps during optimization of a representative “6:6” NP system (${N}_{1}={N}_{2}=6$) whose globally stable structure is the square-packed bilayer obtained earlier through CGMD simulations of polymer-grafted NPs in a polymer bilayer^21^. The entire optimization required only 33 MC steps.

### Structural phases obtained from equal-sized NPs

**Fig. 2:**
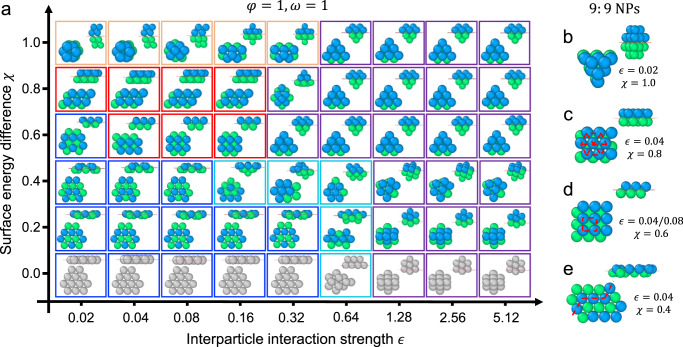
Structural phases of anti-symmetric NPs ($\varphi=1$ and $\omega=1$) predicted from optimization. **a** Phase diagram of structures obtained from a binary 6:6 NP system plotted with respect to interparticle interaction strength $\epsilon$ and surface energy difference $\chi$. The two NP species are depicted by blue and green spheres when they interact differently with the fluids ($\chi \ne 0$) and gray spheres when they interact similarly ($\chi=0$). The blue, red, orange, and purple boxes mark monolayers, bilayers, quasi-globular and globular clusters. Morphologies intermediate to monolayer and globular phases are enclosed by cyan boxes. **b**–**e** Larger scale configurations (9:9 NPs) of four phases seen in (**a**) quasi-globular cluster (**b**), hexagonally ordered bilayer (**c**), square-ordered bilayer (**d**), and hexagonally ordered ridged monolayer (**e**). Source data are provided as a Source Data file.

We first explored the assembly structures formed by a $6:6$ system of “anti-symmetric” NPs, where the two species of NPs have the same size ($\varphi=1$) and the same extent of preference for the opposite fluid layers ($\omega=1$). Figure 2a presents a “phase diagram” of globally stable structures obtained by our optimization approach for different combinations of interparticle interaction strengths $\epsilon$ and relative miscibility values $\chi$. When both NP species interact identically with the two fluids ($\chi=0$), the problem reduces to an optimization of a single species of NPs. As expected, such NPs reside symmetrically at the interface and form hexagonally packed monolayers when interfacial forces dominate interparticle interactions ($\epsilon \, < \,0.32$). This arrangement allows the NPs to occlude the maximum possible area of the interface and gain as many interparticle interactions as possible by a planar array of particles. As $\chi$ becomes larger, the hexagonal monolayer becomes more corrugated (structures enclosed by blue boxes) because the two sets of NPs prefer to stay slightly above and below the interfacial plane. The observed ridged pattern of upward- and downward-displaced NPs allows them to minimize the separation distance between non-neighboring NPs so that they can gain more energy from long-range interparticle interactions.

Bilayers composed of two interdigitated layers of ordered NPs begin to appear upon further increase in $\chi$ in this regime dominated by interfacial forces. These bilayer structures, shown in red boxes, arise due to NPs attempting to gain more energy through their interactions with NPs of the opposite layer while trying to retain as much as possible their preferred interfacial position. A hexagonally ordered bilayer forms at $\chi=0.8$ in which the two layers of NPs are located at $\left\langle z\right\rangle=\pm \!2.50\sigma$ relative to the interfacial plane, where $\left\langle \cdot \right\rangle$ denotes ensemble average over all NPs in each layer. From Eqs. (1), (5), and (6), the preferred interfacial position of such NPs without interparticle interactions is ${z}_{{{{{{\rm{m}}}}}}}=\pm \chi R=\pm 2.4\sigma$ (NP radii $R=3\sigma$), close to their eventual position in the assembled structure. Thus, the NPs sacrifice little interfacial energy in forming the hexagonal bilayer. However, at $\chi=0.6$, for which ${z}_{{{{{{\rm{m}}}}}}}=\pm 1.8\sigma$, the NPs prefer to instead form a square-ordered bilayer that incurs a lesser interfacial energy penalty, as this structure requires the NPs to be less displaced ($\left\langle z\right\rangle=\pm 2.16\sigma$) from their preferred positions ($\pm 1.8\sigma$) compared to the hexagonal bilayer ($\left\langle z\right\rangle=\pm 2.50\sigma$). In both cases ($\chi=0.6$ and $0.8$), the incurred losses in interfacial free energy are easily compensated by the interparticle interaction energy gained through the formation of bilayers.

At $\chi=1$, where ${z}_{{{{{{\rm{m}}}}}}}=\pm R$ and $\triangle F\left({z}_{{{{{{\rm{m}}}}}}}\right)=0$ (from Eq. 2), the two species of NPs would prefer to fully submerge in the fluid layers they are most compatible with and form individual close-packed globular clusters in their respective layers. However, the NPs could gain additional energy through interactions with NPs in the opposite layer. Indeed, our optimization finds that for $\epsilon\, < \,0.64$ the NPs form quasi-globular clusters in their respective fluid layers (shown in orange boxes), where the clusters present large facets in the direction of the interface to maximize NP-NP contacts between clusters across the interface. Unlike the bilayers discussed above, the interacting layers of NPs avoid interdigitation to avoid highly unfavorable interactions with the opposite fluid.

These results show that when interparticle interactions are weak ($\epsilon \, < \,0.64$), $\chi$ is the primary governing factor underlying all structural transitions. This may also be gleaned from the map of ground-state energies (Supplementary Fig. 3), which displays much stronger variation with $\chi$ than $\epsilon$ at small values of $\epsilon$. The map also reveals that $\epsilon$ becomes the dominating factor when $\epsilon > 0.64$. Indeed, when interparticle interactions dominate interfacial interactions, the NPs form close-packed globular clusters (shown in purple boxes), resembling the truncated triangular dipyramid recognized to be the ground-state configuration of a 12-colloid cluster in the bulk^21^. Interestingly, as $\chi$ varies, the clusters retain their truncated triangular dipyramid configuration but adjust their position and orientation with respect to the interfacial plane so that the NPs can maximize their interactions with the interface without losing interparticle interactions. Sandwiched in between monolayers and globular clusters at small $\chi$ values are hybrid morphologies (shown enclosed by cyan boxes) that arise due to compromise between interfacial and interparticle interactions, so these structures exhibit partly planar and partly globular features.

The system size used so far for exploration is not large enough to establish the true periodicity of some of the observed phases due to edge or finite size effects. For example, the square bilayer obtained from optimization appears with either parallel ($\epsilon=0.04,\,\chi=0.6$) or crossed ($\epsilon=0.08,\,\chi=0.6$) layers of NPs (Fig. 2a); at small $\epsilon$, parallel layers are favored because they can bend at their edges to occlude more interfacial area, and at large $\epsilon$, crossed layers are preferred as they can mediate one extra NP-NP contact compared to their parallel counterparts. Despite these differences, both structures are expected to converge to a perfectly ordered square bilayer in the limit $N\to \infty$. To better reveal particle arrangements expected in macroscopic versions of the predicted monolayer and bilayer structures, we applied our optimization strategy to larger systems of NPs (Supplementary Fig. 4), which reduces edge effects and allows periodicity to appear naturally, without imposition of periodic boundaries. Figure 2b–e presents the key structures we achieved from this analysis. We find that the ambiguous conjoined quasi-globular clusters predicted at $\chi=1$ now form more defined hexagonally close packed clusters in the two layers that continue to interact with each other across the interface via their large facets without NP interdigitation (Fig. 2b and Supplementary Fig. 4a). The hexagonal bilayer structure obtained earlier retains its morphology (Fig. 2c and Supplementary Fig. 4b), while the conditions that led to the two variants of square bilayer structures reported earlier now yield the same square-ordered bilayer (Fig. 2d and Supplementary Fig. 4c). Lastly, the punctuated ridges observed in the hexagonal monolayer structures obtained at small $\chi \, > \,0$ take form and lead to zigzag patterns, arguably to maximize the long-ranged attraction between NPs as indicated earlier (Fig. 2e). In the limit of $N\to \infty$, we expect that the planar and ridged monolayer structures would continue to grow into 2D AB-type hexagonally arranged superlattices, the bilayer structures would form 2D AB-type superlattices with square or hexagonal arrangements, and the quasi-globular clusters and the truncated triangular dipyramid would both evolve into 3D fcc or hcp superlattices.

**Fig. 3:**
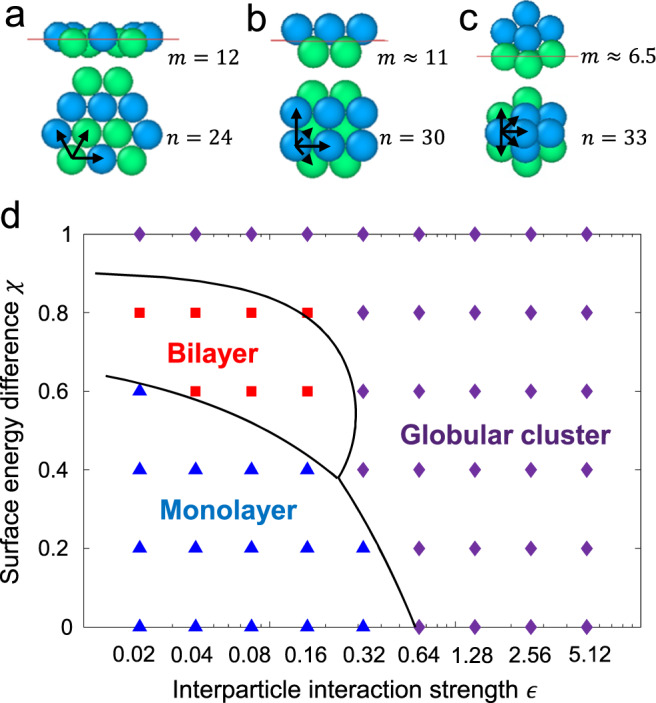
Analytical model for predicting phase coexistence boundaries. **a**–**c** Representative monolayer (**a**), bilayer (**b**), and globular cluster (**c**) phases showing effective numbers of NPs at equilibrium positions $m$ and number of NP-NP contacts $n$ in each structure. Arrows indicate contacts mediated by the chosen NP. **d** Phase boundaries predicted by the model are shown as solid black lines. The structural phases obtained through optimization (from Fig. 2a) are shown by symbols.

The observed transitions in assembly morphology can be described using a simple phenomenological model (see Methods for model derivation). In this model, the total free energy (normalized by $\pi {R}_{1}^{2}\gamma$) of a NP structure is given by the sum of its interfacial and interparticle interaction energies and approximated using $-m{\left(1-\chi \right)}^{2} -n\epsilon$, where $m$ is the effective number of NPs in the structure that maintain their equilibrium positions (${z}_{{{{{{\rm{m}}}}}}}$) and $n$ is the total number of NP-NP contacts exhibited by the structure. By determining the values of $m$ and $n$ for the obtained globally stable clusters (Fig. 3a-c), one can determine the $\chi$- and $\epsilon$-dependent free energies of the monolayer, bilayer, and globular phases, which we denote by ${F}_{{{{{{\rm{m}}}}}}},$ ${F}_{{{{{{\rm{b}}}}}}}$, and ${F}_{{{{{{\rm{g}}}}}}}$. The coexistence boundaries between the three phases can then be determined from phase equilibria conditions ${F}_{{{{{{\rm{m}}}}}}}(\chi,\, \epsilon )={F}_{{{{{{\rm{b}}}}}}}(\chi,\, \epsilon )$, ${F}_{{{{{{\rm{m}}}}}}}(\chi,\,\epsilon )={F}_{{{{{{\rm{g}}}}}}}(\chi,\, \epsilon )$, and ${F}_{{{{{{\rm{b}}}}}}}(\chi,\, \epsilon )={F}_{{{{{{\rm{g}}}}}}}(\chi,\, \epsilon )$ (Supplementary Table 1, Supplementary Fig. 5). The obtained coexistence boundaries (Fig. 3d) show excellent agreement with the earlier phase diagram. The boundaries interestingly point to the existence of a “triple point” where all three phases are in coexistence, suggesting that it may be possible to interconvert NP assemblies between the three phases through slight changes in the parameters $\chi$ and $\epsilon$ about this point.

In addition to antisymmetric NPs ($\omega=1$), we also examined the structural phases formed by “asymmetric” NPs, where the two NP species 1 and 2 are still similar in size but exhibit different extents of preference for opposite fluid layers ($\omega \, \ne \, 1$). The phase diagrams of globally stable configurations of equal-size NPs at $\omega=0$, $\omega=0.5$, and $\omega=2$ are presented in Supplementary Figs. 6–8. At $\omega=0$, NP 2 interacts identically with both fluid layers and thus would prefer to stay symmetrically at the interface. At $\omega=0.5$ or $\omega=2$, NP 2 would exhibit a preferred displacement (${z}_{{{{{{\rm{m}}}}}}}$) that is half of or twice than that of NP 1. We find that most of the structures formed by asymmetric NPs are already revealed in the phase diagram of antisymmetric NPs (Fig. 2a). The reason is because the NP arrangement in the monolayer and bilayer phases is primarily determined by the relative displacement (in the *z* direction) of the two types of NPs, which is given by $\chi (1+\omega )R$ (from Eqs. (1), (5), and (6)) and has been explored via changes in $\chi$, and the structures of the globular phases are mainly determined by the interaction strength $\epsilon$.

### Structural phases obtained from different-sized NPs

Motivated by the uniqueness of some of the layered phases obtained with equal-sized NPs, we explored structures formed by unequal-sized NPs. We examined a $6:6$ binary system of NPs and considered different NP size ratios ($\varphi=5/6,\, 2/3,\, 1/2,$ and $1/3$) holding $\omega$ fixed at a value of $1$ given that this parameter only weakly impacted NP phase behavior. The phase diagrams corresponding to the four size ratios are shown in Supplementary Figs. 9–12, revealing a rich variety of assembly architectures. Like equal-sized NPs, the unequal-sized NPs also form monolayers at small $\epsilon$ and $\chi$, bilayers at small $\epsilon$ but large $\chi$, and globular structures at large $\epsilon$. While the globular structures exhibit compact close-packed morphologies expected to grow into 3D binary crystals akin to those formed in the bulk^42^, the monolayers and bilayers are distinct from those found in bulk solution and from those formed with equal-sized NPs. Therefore, we focused on exploring larger-scale configurations of these monolayer and bilayer phases (superlattices), which display unusual particle arrangements that would be particularly relevant to plasmonic, magnetic, optical, and electronic applications. The obtained configurations were found to be quite robust to deviations in the number ratio of the two NP species (used during structure optimization) from the particle stoichiometry stipulated by superlattice periodicity (Supplementary Fig. 13).

**Fig. 4:**
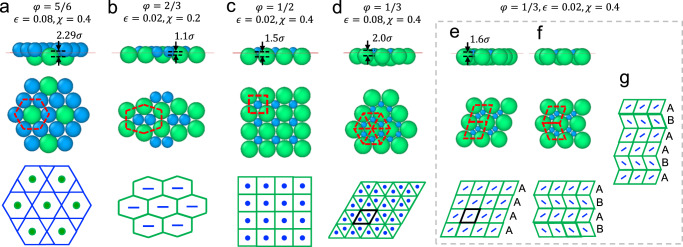
Monolayer BNSLs obtained at different NP size ratios. **a** AB_3_-type BNSL with Kagome-hexagonal order of NPs formed at $\varphi=5/6$. **b** AB-type BNSL with strained honeycomb order at $\varphi=2/3$. **c** AB-type BNSL with square order at $\varphi=1/2$. **d** AB_2_-type BNSL with triangular order at $\varphi=1/3$. **e**–**g** AB_2_-type BNSL with rhombic order showing two equivalent ground-state configurations at $\varphi=1/3$ (**e**, **f**). Random stacking of these two configurations should lead to many possible large-scale hybrid arrangements; one such arrangement is shown for illustration (**g**). Larger-scale patterns formed by the two NPs are shown schematically at the bottom. Black lines mark the unit cells. Source data are provided as a Source Data file.

We begin by discussing the monolayers formed at small $\chi$ values by binary NP systems of slightly different sizes ($\varphi=5/6$). Despite the size difference, we find that the NPs organized themselves into rough hexagonal order to maximize interparticle interactions (Supplementary Fig. 9). At $\chi=0.4$ and $\epsilon=0.08$, however, the NPs form a perfectly hexagonally ordered monolayer in which small NPs form a Kagome sublattice whose voids are filled with large NPs that form a hexagonal sublattice (Fig. 4a). At this $\chi$ value, both NP types prefer to be displaced away from the interfacial plane, with an ideal (equilibrium) spacing of $\chi \left({R}_{1}+{R}_{2}\right)=0.4\times \left(3\sigma+2.5\sigma \right)=2.2\sigma$ between the two sets of NPs. For the small NPs to form perfect hexagons around the large NPs, geometric arguments suggest that the two types of NPs need to be displaced by a distance of $2.29\sigma$ (Supplementary Fig. 14a). As this value is very close to the ideal spacing between the two NP types, the particles sacrifice little interfacial energy to attain the observed close-packed hexagonal arrangement of small and large NPs, which allows the NPs to gain substantial interparticle interaction energy. A larger periodic lattice of such NPs is schematically shown in Fig. 4a to illustrate how these monolayer clusters are expected to continue to grow into a 2D AB_3_-type NP superlattice.

Reducing the size ratio to $\varphi=2/3$ causes the NPs to form stretched versions of hexagonally ordered monolayers (which become especially ordered at $\chi=0.2$), where the large NPs form a strained honeycomb lattice around dimers of small NPs (Fig. 4b). Given that the $1.1\sigma$ spacing observed between the two NP types in this stretched monolayer is also very close to their ideal spacing of $\chi \left({R}_{1}+{R}_{2}\right)=0.2\times \left(3\sigma+2\sigma \right)=1.0\sigma$ (Supplementary Fig. 14b) means that this structure, like the monolayer of Fig. 4a, allows the NPs to gain more interparticle interactions without sacrificing much interfacial energy. This quasi-hexagonal arrangement of NPs is expected to grow into an AB-type 2D superlattice. Another AB-type monolayer is formed at $\varphi=1/2$ in which the large and small NPs form intertwined square sublattices (Fig. 4c). The spacing observed between the two types of NPs in this square arrangement is $1.5\sigma$ (Supplementary Fig. 14c), which is slightly less than the ideal spacing of $\chi \left({R}_{1}+{R}_{2}\right)=0.4\times \left(3\sigma+1.5\sigma \right)=1.8\sigma$. Therefore, NPs sacrifice a small amount of interfacial energy to gain the higher number of interparticle interactions afforded by the square arrangement.

Two distinct varieties of AB_2_-type monolayers form at $\varphi=1/3$ and $\chi=0.4$. The first is a triangular-ordered monolayer formed by relatively strongly interacting NPs ($\epsilon=0.08$), where the large NPs form a hexagonally close-packed monolayer sublattice and the small NPs occupy the interstices formed between the large NPs (Fig. 4d). The other is a rhombic-ordered monolayer formed by weakly interacting NPs ($\epsilon=0.02$), where the large NPs arrange into rhombuses, and the small NPs form dimers and sit at the interstices of this rhombic sublattice (Fig. 4e). Based on their unit cells (marked by black lines in Fig. 4d, e), the two monolayers appear somewhat similar, as both have an underlying rhombic unit cell. However, a key difference is that the triangular monolayer shows direct contact between large NPs and no contact between small NPs, while the rhombic monolayer shows no contact between large NPs and direct contact between small NPs. Since the interaction strength between NPs is proportional to particle size, the unit cell of a triangular lattice displays more favorable interparticle interaction energy than that of a rhombic lattice. On the other hand, the triangular monolayer displays less favorable interfacial energy than its rhombic counterpart, as the small NPs exhibit more deviation from their ideal position ($\left\langle z\right\rangle \approx 0.8\sigma$ compared to ${z}_{m}=\chi {R}_{2}=0.4\sigma$) in the triangular monolayer (Supplementary Fig. 14d) than in the rhombic monolayer, where the small NPs are located almost at the ideal position (Supplementary Fig. 14e); in both monolayers, the large NPs reside close to their ideal position, as they sacrifice much larger interfacial energy compared to small NPs due to their much larger cross-sectional area. Thus, the triangular monolayer with more favorable interparticle interactions and less favorable interfacial interactions is preferred at large values of $\epsilon$, while the rhombic monolayer is preferred at small $\epsilon$; a simple calculation of energy differences predicts a cut off value of $\epsilon \sim 0.053$ for this transition (see Methods).

Our optimization also revealed another globally stable rhombic configuration of NPs (Fig. 4f) with the same energy as the structure in Fig. 4e. In both monolayers, the large NPs form rhombuses while the small NPs sit at their interstices as dimers. The difference is that in Fig. 4e, all small-NP dimers align parallel to each other, while in Fig. 4f, the dimers in adjacent layers align at an angle of ~$90^\circ$. Thus, the structures could continue to grow into larger scale ${AAAA}$ or ${ABAB}$ layered arrangements (Supplementary Fig. 4h), analogous to the hcp and fcc lattices formed in 3D that have the same packing fraction but different stacking: ${ABABAB}$ for hcp and ${ABCABC}$ for fcc. Interestingly, the NPs could also form hybrid structures with randomly mixed $A$ and $B$ layers, an example of which is illustrated in Fig. 4g.

Several of these predicted monolayer NP superlattices have been successfully realized in experiments using NP size ratios similar to those stipulated by our computational predictions^19,43^, as summarized in Supplementary Fig. 15. For example, the AB-type square arrangement of NPs (Fig. 4c) obtained with 2:1 size ratio was assembled at an air-liquid interface from a mixture of 28.9 nm NaFY_4_:Yb/Er + 13.4 nm Fe_3_O_4_ nanocrystals (NCs)^19^ (Supplementary Fig. 15c). A structure similar to our AB_2_-type triangularly ordered BNSL (Fig. 4d) predicted for a size ratio of 3:1 was assembled at an air-water interface from a mixture of 500 nm and 100 nm polystyrene NPs^44^ (Supplementary Fig. 15d). Even the strained honeycomb (Fig. 4b) and rhombic (Fig. 4e, f) BNSLs predicted here have been experimentally realized, albeit using LaF_3_ nanodisks and CdSe/CdS nanorods^43^ (Supplementary Fig. 15g, h), which also have circular cross-sections like our spherical NPs.

**Fig. 5:**
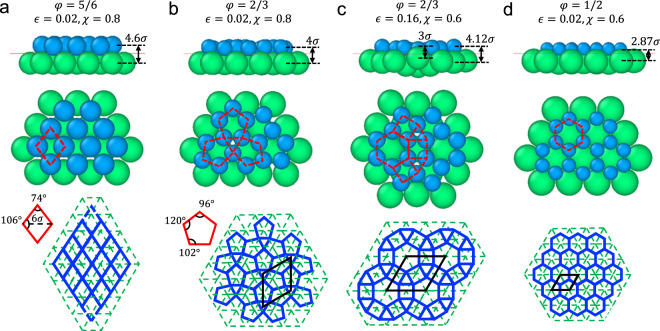
Hybrid bilayer BNSLs formed at different NP size ratios. Large NPs form a hexagonal sheet on which small NPs organize into: **a** quasi-periodic rhombic arrangement at $\varphi=5/6$; **b** periodic tessellation of slender pentagons and triangles at $\varphi=2/3$, yielding an A_3_B_5_-type BNSL; **c** periodic tessellation of hexagons, squares, and triangles at $\varphi=2/3$, yielding an A_4_B_6_-type BNSL; and **d** periodic honeycomb lattice at $\varphi=1/2$, yielding an AB_2_-type BNSL. Larger-scale configurations of the bilayer structures are schematically shown at the bottom with their periodic unit cells marked by black lines. Source data are provided as a Source Data file.

When $\chi \, > \, 0.5$, the NPs prefer to reside further away from the interfacial plane ($|{z}_{m}|$ rises with increasing $\chi$ from Eq. (1)) and their interactions with the interface become weaker (from Eq. (2), |$\triangle F\left({z}_{m}\right)|$ decreases with increasing $\chi$), causing the two sets of NPs to form bilayer configurations (Fig. 5a–d). Thus, interparticle interactions play a stronger role in dictating NP arrangement in bilayer structures than monolayers. As opposed to equal-sized NPs that form a hexagonally packed bilayer at $\chi=0.8$ (Fig. 2c), NPs slightly different in size ($\varphi=5/6$) assemble into a more distinctive hybrid bilayer architecture, where the small and large NP species form layers of different symmetries (Fig. 5a). As the strength of interactions between NPs is proportional to their size, large NPs play a greater role in dictating the ground-state configuration. Consequently, the large NPs preserve their favored hexagonally ordered monolayer arrangement, while the small NPs adjust to the topography of this configuration by forming a “stretched” hexagonal monolayer with a rhombic unit cell of internal angle $106^\circ$ (note that $120^\circ$ angle will lead to regular hexagons). The spacing between the two layers in this hybrid bilayer is $4.6\sigma$ (Supplementary Fig. 16a), close to the ideal value of $\chi \left({R}_{1}+{R}_{2}\right)=0.8\times \left(3\sigma+2.5\sigma \right)=4.4\sigma$, so the NPs sacrifice little interfacial energy to form this hybrid architecture. This structure in which small NPs sit at the interstices of the hexagonal lattice of large NPs also allows the small NPs to maximize their interactions with the large NPs while still allowing for enough contacts amongst themselves. We note however that the rhombic lattice of small NPs is inherently incompatible with the hexagonal lattice of large NPs, as we show schematically in Fig. 5a. Thus, small NPs sitting perfectly at the interstices of large NPs at one location will eventually lose this registry, leading to the formation of grain boundaries at regions of extreme incongruence (Supplementary Figs. 16a, 4d).

Reducing the size ratio to $\varphi=2/3$ yields another intriguing hybrid bilayer architecture (at $\chi=0.8$, $\epsilon=0.02$), where the small NPs form a pentagonal lattice (Fig. 5b) atop a hexagonal lattice of large NPs (also see Supplementary Figs. 16b, 4e). Pentagonal arrangements are rarely found in crystal structures because regular pentagons (with a $108^\circ$ internal angle) cannot form periodic tessellations (Supplementary Fig. 16b). However, in our bilayer, the pentagons are slender, with one angle of $96^\circ$, two angles of $120^\circ$, and two angles of $102^\circ$. This distortion not only enables the small NPs to gain more interfacial energy (as the spacing between the two layers is close to their ideal value of $\chi \left({R}_{1}+{R}_{2}\right)=0.8\times \left(3\sigma+2\sigma \right)=4\sigma$; see Supplementary Fig. 16b), but it also allows the NPs to form a periodic pentagonal arrangement. Three tail-to-tail connected pentagons enclosing a triangle in the center can repeat themselves on the hexagonal lattice formed by the large NPs. Indeed, one $96^\circ$ and two $102^\circ$ inner angles of the three pentagons along with the $60^\circ$ inner angle of the enclosed triangle add up to $360^\circ$ and produce one closed node in the lattice, and three $120^\circ$ inner angles of the three connected pentagons produce another kind of node. As shown in the larger scale structure in Supplementary Fig. 4e and illustrated in Fig. 5b, this arrangement is expected to continue to grow into a 2D A_3_B_5_-type superlattice.

For the same size ratio, reducing $\chi$ to 0.6 and increasing $\epsilon$ to 0.16 causes the NPs to form an equally intriguing hybrid bilayer structure comprised of a repeating pattern of hexagonal, square, and triangular motifs (Fig. 5c, also see Supplementary Figs. 16c, 4f). The large NPs still form a hexagonal lattice, but their displacement from the interfacial plane is uneven—large NPs enclosed by hexagons of small NPs stay closer to the interface than those enclosed by squares (side view, Fig. 5c). Analysis of NP spacings reveals that the small- and large-NP layers are spaced $3\sigma$ and $4.12\sigma$ apart in the hexagonal and square motifs (Supplementary Fig. 16c). Although the hexagonal motif allows the NPs to gain the most interfacial energy (ideal spacing between the two NP types is also $3\sigma$), the small NPs are unable to form a periodic hexagonal arrangement, as this arrangement would be incompatible with the underlying hexagonal lattice of large NPs (Supplementary Fig. 16c). As a result, the NPs instead adopt the multi-motif tessellation, which not only allows the NPs to gain more contacts by forming a periodic arrangement, but also maximize the appearance of hexagonal motifs in the arrangement. This arrangement leads to a 2D A_4_B_6_-type NP superlattice (Fig. 5c and Supplementary Fig. 4f).

Lastly, a periodic honeycomb lattice is formed at a size ratio of $\varphi=1/2$ (Fig. 5d and Supplementary Fig. 4g). Here, small NPs occupy the interstices of three large NPs, culminating in an 2D AB_2_-type NP superlattice. This honeycomb lattice looks similar to the triangular lattice obtained earlier with $\varphi=1/3$ (Fig. 4d and Supplementary Fig. 14d), except that the small NPs in the honeycomb lattice contact each other due to their larger size, leading to closed hexagons. In addition, the spacing observed between the two NP types ($2.87\sigma$) in the honeycomb lattice is quite close to their ideal value of $\chi \left({R}_{1}+{R}_{2}\right)=0.6\times \left(3\sigma+1.5\sigma \right)=2.7\sigma$, implying that the small NPs can gain significant interparticle interactions without sacrificing much interfacial energy.

Among the bilayer NP superlattices reported here, the 2D AB_2_-type honeycomb lattice (Fig. 5d) is the only structure that has been experimentally realized, using polymer-functionalized 5 nm and 10 nm gold NPs ($\varphi=1/2$, in agreement with our predictions) assembled at an air-water interface^45^, and 210 nm and 420 nm oppositely charged polystyrene NPs (also $\varphi=1/2$) obtained via spin-coating method^46^ (Supplementary Fig. 15b). As far as we know, the 2D A_3_B_5_-type (Fig. 5b) and A_4_B_6_-type (Fig. 5c) superlattices predicted here have never been reported.

## Discussion

This work explores the repertoire of 2D superlattices formed by a binary system of NPs at a fluid-fluid interface. Our approach of using analytical models for interparticle and interfacial interactions and an efficient Monte Carlo optimization algorithm allows us to rapidly determine the ground-state configurations of NPs trapped at an interface. By exploring NP systems of varying size ratios, interparticle interaction strengths, and miscibility differences with the two fluids, we discover a range of NP monolayer, bilayer, and globular phases. The formation of these phases is shown to arise from a competition between interfacial and interparticle interactions, and the phases boundaries are captured by an analytical model based on the effective number of NPs occupying their preferred interfacial position and the number of NP-NP contacts in the structures. By analyzing particle arrangements in large-scale configurations of these phases, we uncover a rich variety of mono- and bilayer NP superlattices. It has been demonstrated that binary NP superlattices possess plasmonic, magnetic, optical, and electronic properties distinct from those exhibited by individual NPs^18,47–49^. Such collective properties depend on the symmetry and interparticle spacing of the superlattices. The diverse superlattices found in this work, combined with an understanding of their assembly revealed here, should offer opportunities for researchers to fabricate such superlattices and explore their properties and applications. Several studies have shown that NP films assembled at fluid interfaces can be successfully lifted off the interface and deposited onto a substrate^19,50–52^.

While some of the predicted structures have been experimentally realized^19,43,44,46,53,54^ (Supplementary Fig. 15), others such as the A_3_B_5_- and A_4_B_6_-type BNSLs will need to be validated and could be worthy candidates of future experimental efforts. To this end, the dimensionless parameters explored in this work would need to be linked to experimental conditions. To illustrate this process, we consider the system of silica ($2$) and silver ($1$) NPs at an air ($2'$)-water ($1'$) interface of surface tension $\gamma=72.2$ mJ/m^2^ ^55^. The surface energy of silica in air (${\gamma }_{2{2}^{\prime}}$) and water (${\gamma }_{2{1}^{\prime}}$) is 287 mJ/m^2^ and 340 mJ/m^2^ ^55,56^, which yields $\triangle {\gamma }_{2}\equiv {\gamma }_{2{1}^{\prime}}-{\gamma }_{2{2}^{\prime}}=53$ mJ/m^2^. The difference in the surface energy of silver with air and water can be estimated from the contact angle of water on silver via Young’s equation: $\triangle {\gamma }_{1}\equiv {\gamma }_{1{2}^{\prime}}-{\gamma }_{1{1}^{\prime}}=\gamma \, {{{{{\rm{cos }}}}}}\,{64}^{{{{{{\rm{o}}}}}}}\, \approx \,31.7$ mJ/m^2^ ^57^. From Eqs. 5 and 6, we then obtain $\chi=0.44$ and $\omega=1.67$. The remaining parameter $\epsilon$ can be estimated from the measured Hamaker constant of silver in water, as given by 61.6 zJ^58^. As shown in Methods, this value yields a vdW attraction energy of ${\varepsilon }_{11}=$135 zJ between two $R=$ 3 nm silver NPs, and then $\epsilon=0.066$ from Eq. (4). Therefore, according to our phase diagram, this example system would have a good chance of forming the AB-type square bilayer at size ratio $\varphi=1$ (Fig. 2) and forming the AB_2_-type honeycomb bilayer at size ratio $\varphi=1/2$ (Supplementary Fig. 11**;** Fig. 5d).

Although we focused on binary NP structures, this work could easily be extended to ternary and quaternary NP superlattices. The additional NP species of distinct sizes or surface chemistry would introduce additional functionalities into the NP composite. However, fabrication of such multi-component NP superlattices is experimentally challenging and only a handful of ternary superlattices have so far been reported^19,43,59^. Recent MD simulations have shown that cubic NPs can be assembled into distinct 1D and 2D periodic architectures at fluid interfaces by taking advantage of their ability to tune the orientation of anisotropic NPs^60^. Superlattices of more complex-shaped NPs at interfaces could also be explored with our optimization strategy. The main challenge here would be the analytical treatment of interparticle and interfacial interactions, required for efficient discovery of assembly structures. Recent studies have indeed started to address these challenges, especially for nanocubes and other faceted NPs^61,62^. Our approach for searching for NP superlattices could be further improved by integrating strategies used in crystal structure prediction^63,64^, where lattice parameters, including angles and lengths of lattice vectors could be included as additional variables in the optimization. This may reduce the computational cost of optimization, as the large-area NP superlattices could be replaced by a single unit cell with periodic boundaries to avoid edge effects. While our model is most applicable to NPs much larger than the roughness of the interface and interparticle interactions dominated by vdW forces, our model can be readily extended to NP systems exhibiting other kinds of attractive interactions, such as those mediated by surface ligands or depletion forces. It is possible that the interactions between such NPs could deviate from the Lorentz-Berthelot rule assumed here, so the strength of inter-species attraction is no longer intermediate to the strengths of intra-species attraction, which could lead to other kinds of NP assemblies (Supplementary Fig. 17). In some cases, the surface of NPs is grafted with long polymer chains to assist in their assembly, where multibody effects become important^65^. Our optimization approach would then need to be combined with many-body potentials to uncover the more irregular, anisotropic NP assemblies expected to form as a result of multibody effects^21^.

## Methods

### Model for interparticle interactions

While NPs are typically grafted with molecules or polymer chains and interact with each other via a combination of solvent-dependent van der Waals (vdW) forces, solvent-mediated depletion forces, and graft-mediated interactions, our model treats the two types of NPs as spheres of radii ${R}_{1}$and ${R}_{2}$ that interact with each other, independent of the surrounding fluid, via a shifted Lennard-Jones (LJ) pairwise potential:

7 $${U}_{{ij}}(r)=4{\varepsilon }_{{ij}}\left[{\left(\frac{\sigma }{r-{\triangle }_{{ij}}}\right)}^{12}-{\left(\frac{\sigma }{r-{\triangle }_{{ij}}}\right)}^{6}\right]$$

where ${ij}$ = 11, 22 and 12 denotes interactions between like and unlike pairs of NPs, $r$ is the separation distance between the NPs, $\sigma$ represents an intrinsic length scale of the system, and ${\triangle }_{{ij}}={R}_{i}+{R}_{j}-\sigma$ is a distance shift that prevents NPs from penetrating each other. We find that the resulting potential captures well the vdW interactions between small colloidal NPs (Supplementary Fig. 1). The interaction strengths were assumed to be proportional to NP radii, consistent with the size scaling of vdW interactions between spherical colloids^38^, so that

8 $${\varepsilon }_{22}=\frac{{R}_{2}}{{R}_{1}}{\varepsilon }_{11},\,{\varepsilon }_{12}=\sqrt{{\varepsilon }_{11}{\varepsilon }_{22}}=\sqrt{\frac{{R}_{2}}{{R}_{1}}}{\varepsilon }_{11}$$

where we used Lorentz-Berthelot rules to infer the strength of interactions between unlike particles. The above model thus captures both the size-dependence in the depth of the interparticle interaction potential well and its narrower width (relative to particle size) compared to that of interatomic LJ potentials.

### Model for interfacial free energy

We used an analytical model we recently developed^21^ to describe the interfacial free energy of a surface-functionalized NP trapped at the interface (Supplementary Fig. 2). According to the model, the interfacial free energies of the two species of NPs (relative to their reference bulk values in their favored fluid layer) as a function of their normal position $z$ from the interface are given by

9 $$\triangle {F}_{1}(z)=\left\{\begin{array}{ccc}0 \hfill & z\le -{R}_{1}\hfill\\ -\pi \left({R}_{1}^{2}-{z}^{2}\right)\gamma+2\pi {R}_{1}\left({R}_{1}+z\right)\triangle {\gamma }_{1} \hfill & \left|z\right|\, < \, {R}_{1}\hfill\\ 4\pi {R}_{1}^{2}\triangle {\gamma }_{1} \hfill & z\ge {R}_{1}\hfill\end{array}\right.$$

10 $$\triangle {F}_{2}(z)=\left\{\begin{array}{ccc}4\pi {R}_{2}^{2}\triangle {\gamma }_{2} \hfill & z\le -{R}_{2}\\ -\pi \left({R}_{2}^{2}-{z}^{2}\right)\gamma+2\pi {R}_{2}\left({R}_{2}-z\right)\triangle {\gamma }_{2}\hfill & \left|z\right|\, < \,{R}_{2}\hfill\\ 0\hfill & z\ge {R}_{2}\hfill\end{array}\right.$$

where $\gamma$ is the surface tension between the two fluids, and $\triangle {\gamma }_{1}\equiv {\gamma }_{1{2}^{\prime}}-{\gamma }_{1{1}^{\prime}}\ge 0$ and $\triangle {\gamma }_{2}\equiv {\gamma }_{2{1}^{\prime}}-{\gamma }_{2{2}^{\prime}}\ge 0$ are the differences in the surface energies of NP 1 and 2 with the two layers. When NPs intersect with interfacial plane ($\left|z\right|\, < \, {R}_{i}$), the free energy changes arise from two contributions: free energy gain due to the NP occluding part of the interface (first term) and free energy loss due to the unfavorable interactions between the NP and the incompatible layer (second term). The equilibrium position ${z}_{{{{{{\rm{m}}}}}}}$ of the NP (in the absence of interparticle interactions) can then be determined by $\partial \triangle {F}_{i}/\partial z{|}_{{z}_{{{{{{\rm{m}}}}}}}}=0$, yielding Eq. (1) and (2).

### Optimization approach

We used the basin-hopping Monte Carlo (BHMC) global optimization method for locating ground-state configurations of NPs at the fluid-fluid interface^39–41^, that is, global minimum of the total energy of the system as given by

11 $${E}_{{{{{{\rm{tot}}}}}}}\left({{{{{{\bf{r}}}}}}}^{N}\right)=\mathop{\sum }\limits_{i=1}^{N-1}\mathop{\sum }\limits_{j=i+1}^{N}{U}_{I\left(i\right)I(j)}({r}_{{ij}})+\mathop{\sum }\limits_{i=1}^{N}{\triangle F}_{I(i)}\left({z}_{i}\right)$$

where ${{{{{{\bf{r}}}}}}}^{N}$ represents the $3N$ coordinates of the NPs and $I$ is a function that determines the identity of the particles (NP 1 or 2). In this algorithm, the original intricate potential energy surface ${E}_{{{{{{\rm{tot}}}}}}}\left({{{{{{\bf{r}}}}}}}^{N}\right)$ is effectively transformed through local minimization into a staircase topography, represented by ${\widetilde{E}}_{{{{{{\rm{tot}}}}}}}\left({{{{{{\bf{r}}}}}}}^{N}\right)={{\min }}\{{E}_{{{{{{\rm{tot}}}}}}}\left({{{{{{\bf{r}}}}}}}^{N}\right)\}$, which is then explored via MC sampling using the Metropolis criterion. As shown in the flow chart (Fig. 1b), the initial seed configuration was randomly generated in a cuboid box in which the interfacial plane was fixed at $z=0$. A sequence of MC moves was then implemented to produce new seed configurations, which were further optimized by using local minimization approaches. In particular, the Polak-Ribiere variant of the conjugate gradient algorithm^66^ was used for local minimization and the inexact line search was performed based on the Wolfe conditions^67,68^. The Metropolis criterion was then used to update the NP configuration, which was then set as the seed structure for the next iteration. The above process was repeated until convergence was reached. While ground-state structures are strictly speaking only valid at zero temperature, differences in vibrational entropy of competing crystalline structures are often small. Thus, structures obtained via energy minimization may also represent minimum free-energy structures at non-zero temperatures.

### New Monte Carlo moves

The MC displacement move implemented in standard BHMC algorithms for particle assembly is designed to search for 3D clusters and is not efficient for searching for the 2D structures typically obtained in interfacial assembly^13,19,21^. Inspired by the work of Rondina and Silva^41^, we introduced 5 additional MC moves into the standard BHMC algorithm for improving the sampling efficiency of NP configurations at interfaces (Fig. 1b):

(i) Displacement move (**D**). This is the standard move employed in the original BHMC algorithm, which randomly displaces NPs around their original positions. The maximum displacements are fixed for all NPs.
(ii) Position-based displacement move (**P**). In this variant of **D**, the magnitude of NP displacement depends on the distance of the NP from the centroid of the cluster^41,69^. This move takes advantage of differences in the energy landscape experienced by NPs at the surface versus core of the clusters.
(iii) Twist move (**T**). This move separates NPs into two categories based on whether they are above or below the interfacial plane. The move then randomly selects one of the two categories and rotates all NPs in that category by a random angle about an axis normal to the interface passing through the cluster centroid.
(iv) Interfacial move (**I**). It usually takes a long “diffusive time” for a NP fully immersed in one liquid to migrate toward the interface. Therefore, this move directly displaces the NP farthest from the interfacial plane to a plane near the interface.
(v) Switch move (**S**). Since both species of NPs exhibit favorable interactions with one fluid layer and unfavorable interactions with the opposite layer, there is a free energy loss when NPs intrude into opposite layers. This move targets pairs of NPs of opposite species that intrude into their corresponding disfavored layer and then switches their type.
(vi) Rotation move (**R**). This move chooses either the NP farthest from the cluster centroid or the NP with the least neighbors and randomly rotates the chosen NP on a circle parallel to the interfacial plane. This move is designed for filling in vacant sites in planar structures.

These moves were placed in a loop sequence of **P-S-T-D-I-S-R-D-R**. Each move in the sequence was executed for a period of MC steps until the move was consecutively rejected by the Metropolis criterion, upon which we switched to the next move in the sequence. We further realized that NPs could get trapped in very deep local minima from which they are unable to escape via the ongoing MC move, resulting in the same structure appearing again and again. To circumvent this, we implemented the ultrafast shape recognition algorithm^70,71^ to identify duplicated NP structures. The BHMC algorithm would then directly jump to the next type of MC move if such duplicated structures are detected in consecutive MC steps. For optimization of large systems, an additional operation was added to the MC cycle, where the current best structure (till that MC step) was taken as the seed structure (instead of the instantaneous structure at that step) after every 2000 MC steps. This is because the current best structure was found to typically have more similarity to the ground-state configuration compared to the instantaneous structure.

### Validation of optimization strategy

First, we adapted our optimization algorithm to the well-known bulk system of Lennard-Jones atom clusters, as there is no previous optimization work on 2D BNSLs at interfaces. Our algorithm was able to accurately locate the known global minima for three representative clusters containing up to 50 atoms^39,72,73^ (Supplementary Fig. 18a). Second, we compared our predicted NP structures against observations from CGMD simulations that studied assembly of polymer-grafted NPs at a polymer-polymer interface^21^. These simulations showed that bare NPs that interact neutrally with both polymer layers assemble into a hexagonally arranged monolayer positioned symmetrically at the interface (Supplementary Fig. 18b, bottom left); our optimization algorithm predicts the same morphology for a single species of neutral NPs, i.e., $\chi=0$ (Supplementary Fig. 18b, top left). When co-assembling two species of grafted NPs with ligands that have different miscibility with the two fluids, a square bilayer was obtained in the CGMD simulations, which is reproduced by our algorithm for the case $\chi=0.6$ where the NPs strongly favor the opposite fluids of the interface (Supplementary Fig. 18b, middle). When the interactions between NPs ($\epsilon=2.56$) are large enough to overwhelm those between the NPs and the interface, the NPs form the same globular structure they form during bulk assembly;^74^ this structure is also correctly reproduced by our optimization algorithm (Supplementary Fig. 18b, right).

### Phenomenological model of phase boundaries

The total free energy of a structural phase is composed of two terms: interfacial energy of the NPs and interparticle interaction energy. Using Eqs. (2) and (5), the interfacial energy (normalized by $\pi {R}_{1}^{2}\gamma$) can be expressed as $-m{\left(1-\chi \right)}^{2}$, where $m$ is the effective number of NPs at their equilibrium positions (${z}_{{{{{{\rm{m}}}}}}}$). As the interactions between NPs are short ranged, the interaction energy between NPs (normalized by $\pi {R}_{1}^{2}\gamma$) can be approximated by $-n\epsilon$ (see Eq. 4), where $n$ is the total number of NP-NP contacts exhibited by the structure. Therefore, the total free energy of a structure (normalized by $\pi {R}_{1}^{2}\gamma$) can be represented by $-m{\left(1-\chi \right)}^{2}-n\epsilon$ .

For monolayer structures in the phase diagram, all particles can sit at their preferred interfacial positions without sacrificing any interparticle interactions, so $m=N=12$, and counting NP-NP contacts in the structure reveals that $n=24$ (Fig. 3a; Supplementary Table 1). For the bilayer structures, $m$ can be determined from the ratio of their total interfacial energy and the minimum value of the interfacial free energy, that is, $-{\left(1-\chi \right)}^{2}$. Based on geometry, the half-spacing between the two layers of NPs in the square and hexagonal bilayers is given by ${z}_{s}=2.16\sigma$ and $2.5\sigma$. These values can be substituted into the Eqs. 9 and 10 to obtain the interfacial energy, and thereby $m$. We find that $m\approx 11$ for the square bilayer and $m\approx 10$ for the hexagonal bilayer at $\chi=0.6$. Furthermore, we obtain $n=30$ for the square bilayer and $n=31$ for the hexagonal bilayer (Fig. 3b; Supplementary Table 1). Lastly, for the globular clusters, specifically the truncated triangular dipyramid structure, $n=33$. Since the globular clusters can adjust their position and orientation with respect to the interface plane as $\chi$ varies, $m$ could change with $\chi$. To this end, we take an averaged value of $m$ for all structures above or below $\chi=0.5$, which give $\bar{m}\, \approx \, 6.5$ for $\chi \, > \, 0.5$ and $\bar{m}\, \approx \,4.5$ for $\chi < 0.5$. (Fig. 3c; Supplementary Table 1). By equating the total energies from pairs of the structural phases, we obtain the following three equations for boundaries between the three structural phases (Supplementary Fig. 5):

- Boundary between monolayers and bilayers (blue line):
  12 $$\epsilon=2{\left(0.72-\chi \right)}^{2}$$
- Boundary between bilayers and globular clusters (red line):
  13 $$\epsilon=-\!2.18{\left(1-\chi \right)}^{2}-12\left(1-0.833\right)\chi+6(1-{0.833}^{2})$$
- Boundary between monolayers and globular clusters (purple line):

14 $$\epsilon=\frac{5.5{\left(1-\chi \right)}^{2}}{9}$$

### Predicting transition between triangular and rhombic monolayers

Since the interaction strength between NPs is proportional to particle size, the unit cell of a triangular lattice (with effectively one contact formed between large NPs) would display more favorable interparticle interaction energy than that of a rhombic lattice (with one contact formed between two small NP). According to Eq. (8), this interaction energy difference is roughly equal to the difference ${\varepsilon }_{11}-{\varepsilon }_{22}={{\varepsilon }}_{11}(1-R_{2}/{R}_{1})$, yielding a dimensionless energy of $2/3\epsilon$ via Eq. (4). On the other hand, the triangular monolayer displays less favorable interfacial energy than its rhombic counterpart, as the small NPs exhibit more deviation from their ideal position ($\left\langle z\right\rangle \approx 0.8\sigma$ compared to ${z}_{m}=\chi {R}_{2}=0.4\sigma$) in the triangular monolayer (Supplementary Fig. 14d) than in the rhombic monolayer, where the small NPs are located almost at the ideal position (Supplementary Fig. 14e). In both monolayers, the large NPs reside very close to their ideal position (${z}_{m}=\chi {R}_{1}=1.2\sigma$), as large NPs sacrifice much larger interfacial energy compared to the small ones due to their $\times 9$ larger cross-sectional area. The interfacial energy difference between the unit cells of the two monolayers can then be estimated by the difference in the interfacial energy of their two small NPs, as given by $2[\triangle {F}_{2}\left(0.8\sigma \right)-\triangle {F}_{2}(0.4\sigma )]$, which yields a dimensionless energy value of $0.0356$ via Eq. 10. When $\epsilon \, > \, 0.053$ (obtained by setting $2/3\epsilon \, >$ $0.0356$), the loss in interfacial energy can be compensated by the gain in interparticle interactions, favoring the formation of the triangular monolayer (Fig. 4d). Otherwise, the NPs prefer to maximize their interfacial energy by forming the rhombic monolayer (Fig. 4e).

### Model for predicting vdW interaction energy of spherical NPs

Assuming that the atoms in the two NPs are of size $\sigma$ and interact via the LJ potential, the vdW energy $W(D)$ between two NPs of radii $R$ separated by a surface-to-surface distance of $D$ $(\ll R)$ is given by $W\left(D\right)={AR}({\sigma }^{6}/2520{D}^{7}-1/12D)$^38^. At the energy minimum (${30}^{-1/6}\sigma$), the vdW attraction is given by $\approx -\!0.126{AR}/\sigma$. Since the Hamaker constant of silica NPs ($\sigma=\,$0.21 nm) in air is 65 zJ^75^, and that of silver NPs ($\sigma=$0.172 nm) in water is 61.6 zJ^58^, the interparticle interaction strength between silica NPs and between silver NPs are given by ${\varepsilon }_{22}=\,$117 zJ and ${\varepsilon }_{11}=\,$135 zJ, using $R=$ 3 nm.

## Supplementary information

Supplementary Information

## Data Availability

The data generated in this study have been deposited in the public GitHub (https://github.com/yilongz393/Tailored-BHMC) without any restrictions. Source data are provided with this paper.

## Source data

Source Data

## Code availability

The codes used in this study have been deposited in the public GitHub (https://github.com/yilongz393/Tailored-BHMC) without any restrictions.
