# Supplementary material for: Discovery of two-dimensional binary nanoparticle superlattices using global Monte Carlo optimization
Source: Nat Commun. 2022 Dec 29;13:7976. doi: 10.1038/s41467-022-35690-8 (PMC9800587; doi:10.1038/s41467-022-35690-8)
Supplement: Supplementary file 1 — Supplementary Information [file 41467_2022_35690_MOESM1_ESM.pdf]

Supplementary Information:

Discovery of Two-Dimensional Binary Nanoparticle Superlattices Using Global Monte Carlo Optimization

Yilong Zhou[1,2] and Gaurav Arya[1*]

[1]Department of Mechanical Engineering and Materials Science, Duke University, Durham, NC 27708, USA

[2]Present address: Materials Science Division, Lawrence Livermore National Laboratory, Livermore, CA 94550, USA

* Corresponding author

Email: gaurav.arya@duke.edu, Phone: +1 (919) 660-5435, Fax: +1 (919) 660-8963

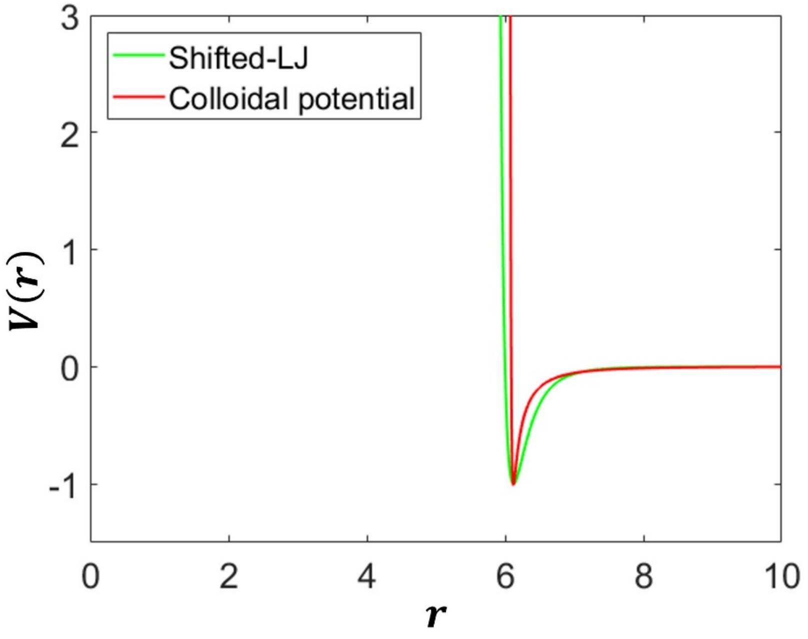

Supplementary Fig. 1 Comparison of the shifted-LJ potential used in this work and the inter-colloidal vdW interaction potential for silica NPs of size $R = 3$ nm. The energies in both potentials are normalized by the magnitude of the potential energy minimum.

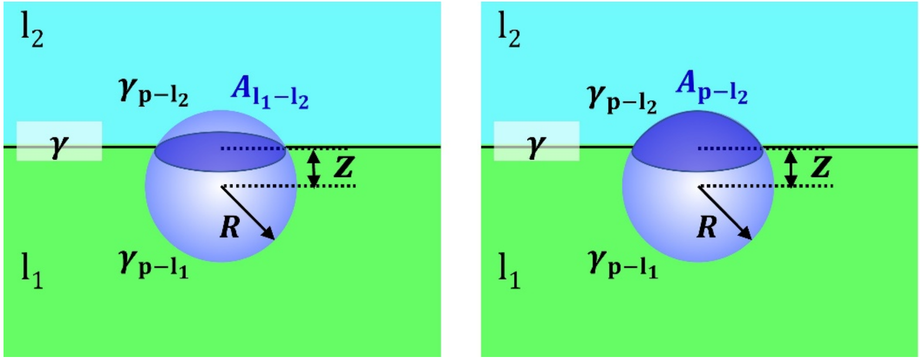

Supplementary Fig. 2 Schematic showing the two contributions to the interfacial free energy of trapping a spherical NP at the interface: the free energy gain due to the NP occluding part of the interface proportional to the circular cross-sectional area $A_{l_1-l_2}$ and the free energy loss due to the unfavorable interactions between the NP and the incompatible fluid (l_2) proportional to the area of the spherical cap A_{p-l_2}. The free energy change associated with bringing a NP from the bulk region of the compatible fluid l_1 to a distance z from the interfacial plane is given by $\Delta F(z) = -A_{l_1-l_2}\gamma + A_{p-l_2}\Delta\gamma_{pl}$, where γ is the interfacial tension between the two liquids and $\Delta\gamma_{pl} \equiv \gamma_{p-l_2} - \gamma_{p-l_1}$ is the difference in the surface energies of the NP with the two fluids. Substituting areas corresponding to a NP of radius *R*, yields $\Delta F(z) = -\pi(R^2 - z^2)\gamma + 2\pi R(R + z)\Delta\gamma_{pl}$. The equilibrium position z_m of the NP can then be determined by setting $\partial\Delta F(z)/\partial z|_{z_m} = 0$, yielding $z_m = -\Delta\gamma_{pl}/\gamma R$ (Eq. 1). The interfacial free energy of the NP at this equilibrium position z_m is then given by $\Delta F(z_m) = -\pi R^2\gamma\left(1 - \Delta\gamma_{pl}/\gamma\right)^2$. In the limit that the NP interacts similarly with the two fluids, i.e., $\Delta\gamma_{pl} = 0$, the NP sits symmetrically at the interface occluding an area of πR^2, so the free energy change is simply equal to this area multiplied by surface tension γ, i.e., $\Delta F(z_m) = -\pi R^2\gamma$. In the limit that the NP interacts similarly with l_2 as l_1 does with it, i.e., $\Delta\gamma_{pl} = \gamma$, the interface gains no free energy from particle occlusion whereas the NP loses free energy due to loss of favorable interactions with l_1. The NP remains in the bulk of l_1, and the free energy change is 0.

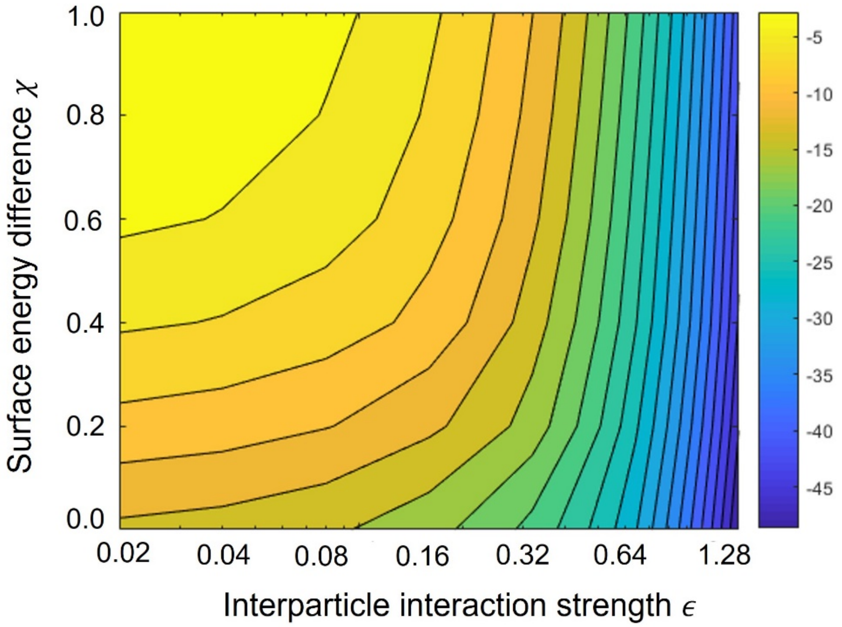

Supplementary Fig. 3 Contour map of the total energy E_{tot} of the minimum-energy NP configurations depicted in Fig. 2*a*.

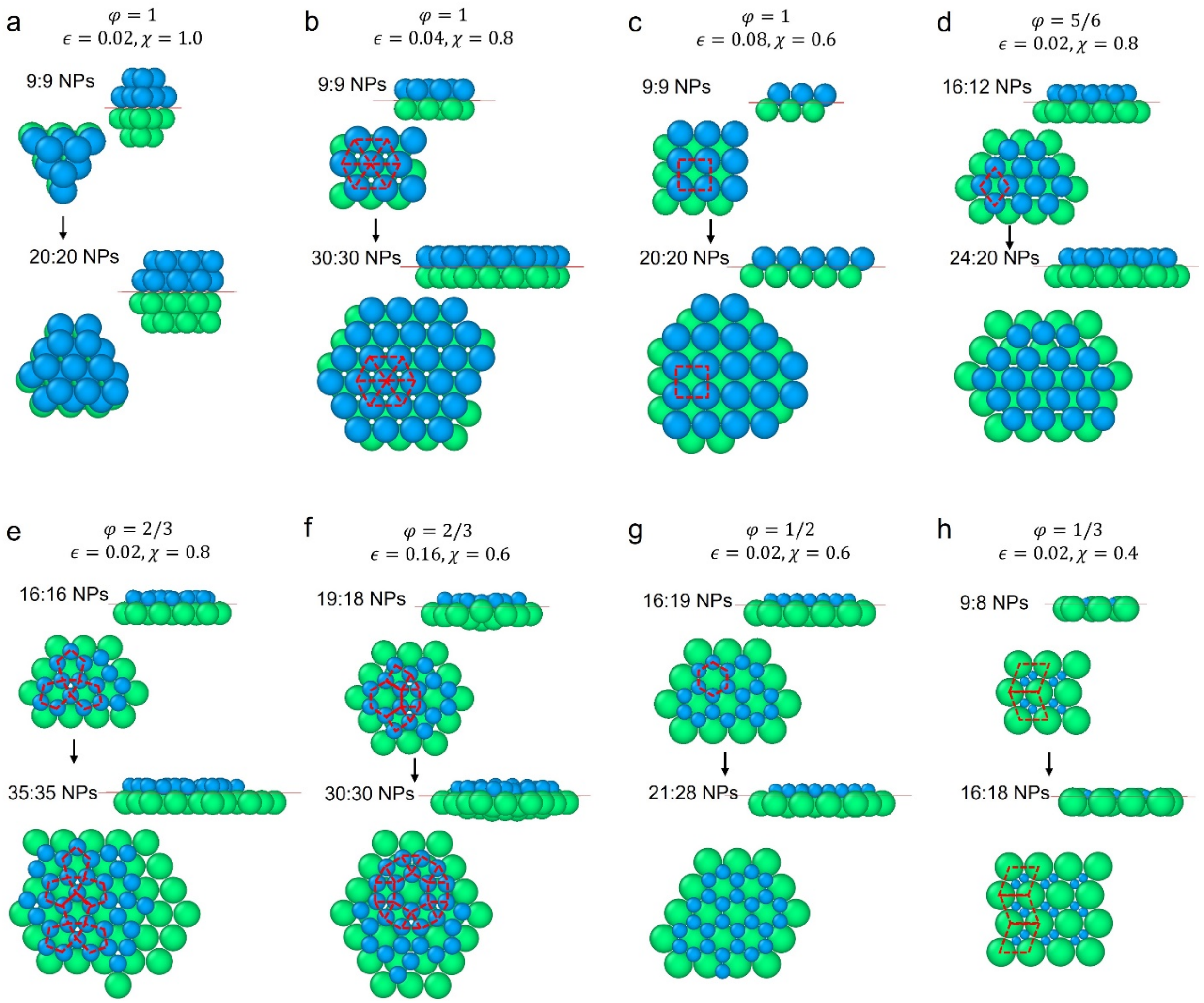

Supplementary Fig. 4 Large-scale NP structures obtained through optimization. **a** Quasi-globular clusters. **b** Hexagonally ordered BNSL. **c** Square-ordered BNSL. **d** BNSL with quasi-periodic rhombic arrangement. **e** BNSL with periodic tessellation of slender pentagons and triangles. **f** BNSL with periodic tessellation of hexagons, squares, and triangles. **g** BNSL with periodic honeycomb lattice. **h** BNSL with rhombic order. Top structures were reproduced from Figs. 2, 4 and 5**,** and bottom structures were obtained with larger number of NPs.

Supplementary Table 1. Comparison of the total energy of NP structures predicted by our analytical model for various monolayer, bilayer, and globular phases.

**Structural Phases**		$\boldsymbol{m}$	$\boldsymbol{n}$	$\boldsymbol{E_{\mathrm{tot}}}$
**SP1**	**SP2**			
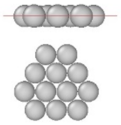	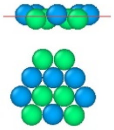	12	24	$-m(1-\chi)^2 - n\epsilon$
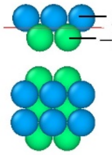	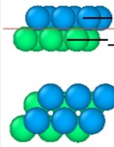	$\frac{12\left\{-\left(1-\left(\frac{z_s}{R}\right)^2\right)+2\left(1-\frac{z_s}{R}\right)\chi\right\}}{-(1-\chi)^2}$<br>(SP1: $z_s = 2.16\sigma$<br>SP2: $z_s = 2.5\sigma$)	SP1: $n = 30$<br>SP2: $n = 31$	
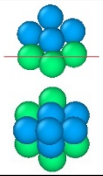	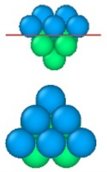	SP1: $\bar{m} \approx 6.5$<br>SP2: $\bar{m} \approx 4.5$	33	

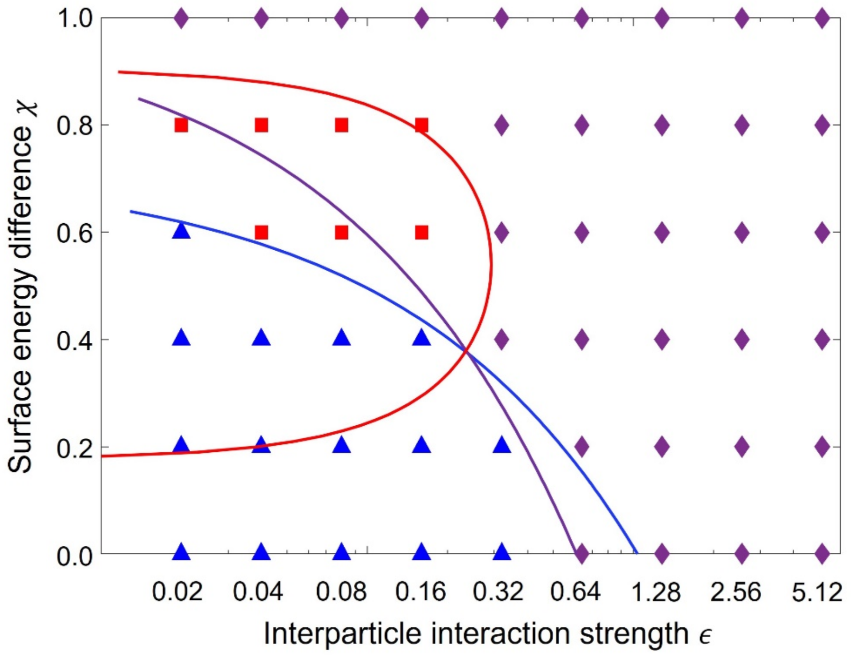

Supplementary Fig. 5 Boundaries between monolayer, bilayer, and globular phases predicted by our analytical model based on the analysis of m and n of the corresponding structures. The boundaries between the monolayer and bilayer phases, the monolayer and globular phases, and the bilayer and globular phases are shown by blue, purple, and red lines, respectively.

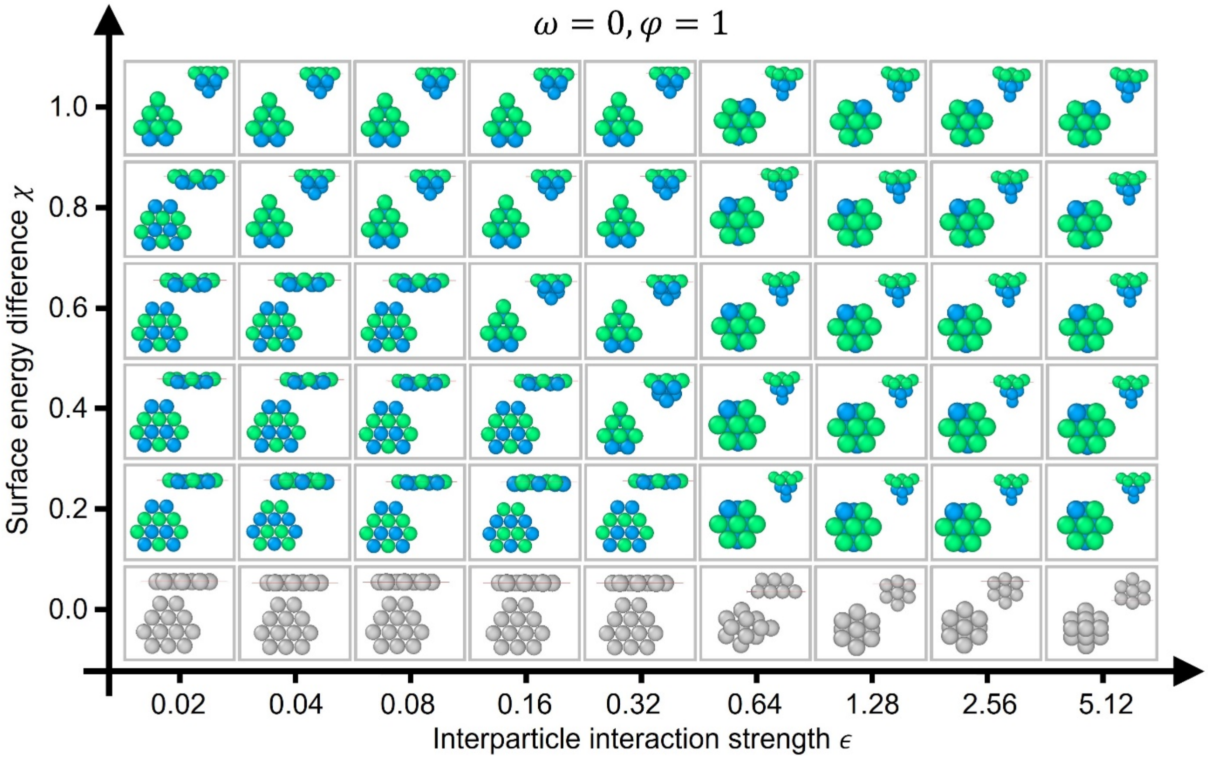

Supplementary Fig. 6 Structural phase diagram of 6:6 NPs (top and side views) at $\omega = 0$ and $\varphi = 1$ with respect to the interparticle interaction strength parameter ϵ and the relative miscibility parameter χ.

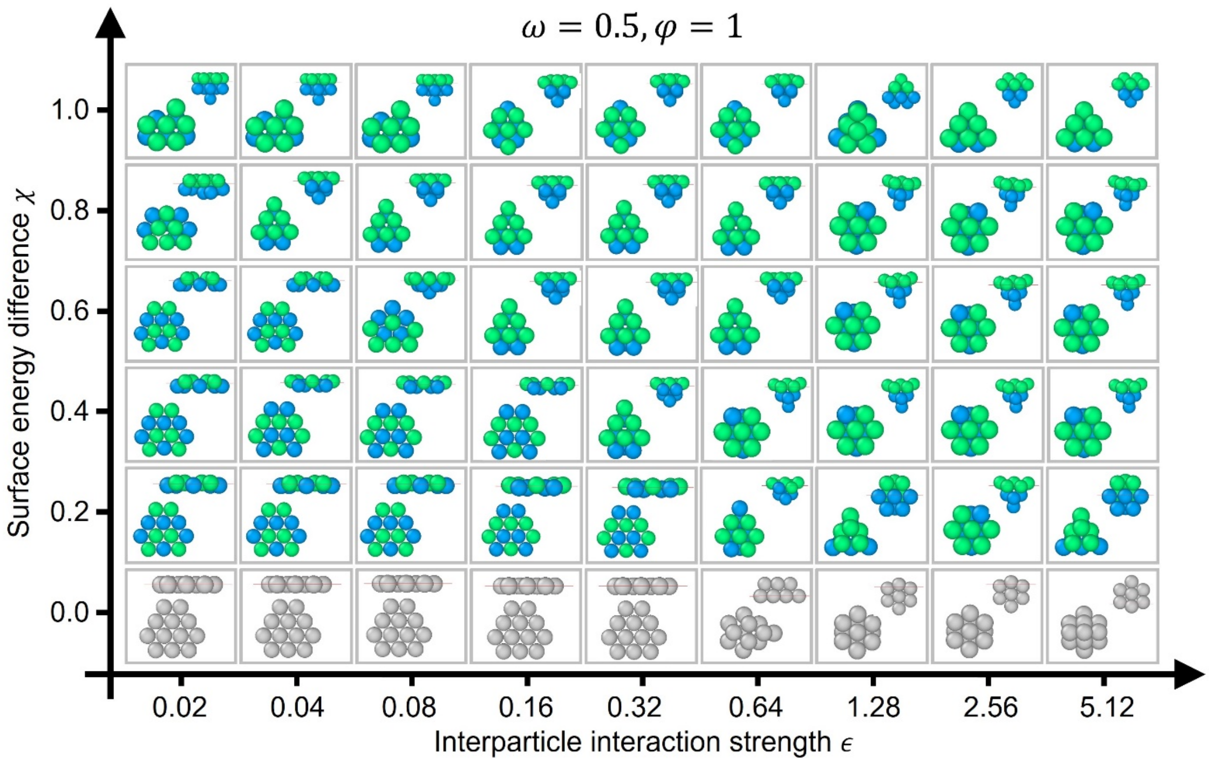

Supplementary Fig. 7 Structural phase diagram of 6:6 NPs (top and side views) at $\omega = 0.5$ and $\varphi = 1$ with respect to the interparticle interaction strength parameter ϵ and relative miscibility parameter χ.

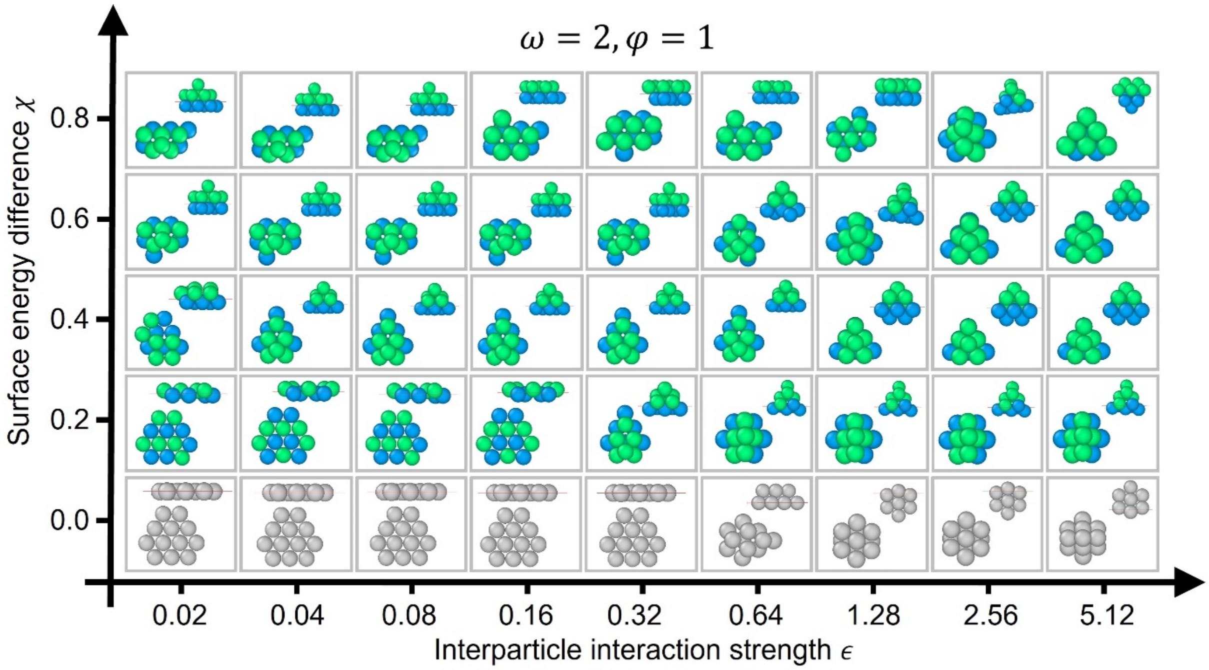

Supplementary Fig. 8 Structural phase diagram of 6:6 NPs (top and side views) at $\omega = 2$ and $\varphi = 1$ with respect to interparticle interaction strength parameter ϵ and relative miscibility parameter χ.

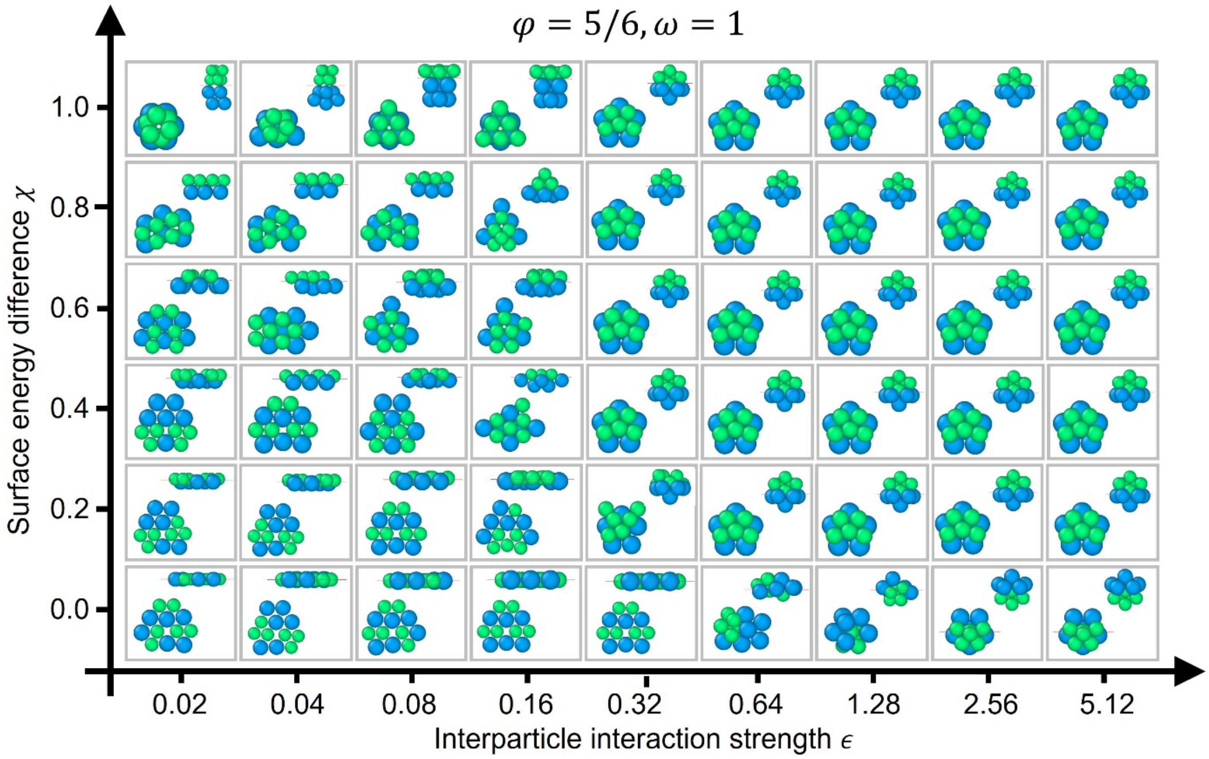

Supplementary Fig. 9 Structural phase diagram of 6:6 NPs (top and side views) at $\varphi = 5/6$ and $\omega = 1$ with respect to interparticle interaction strength parameter ϵ and relative miscibility parameter χ.

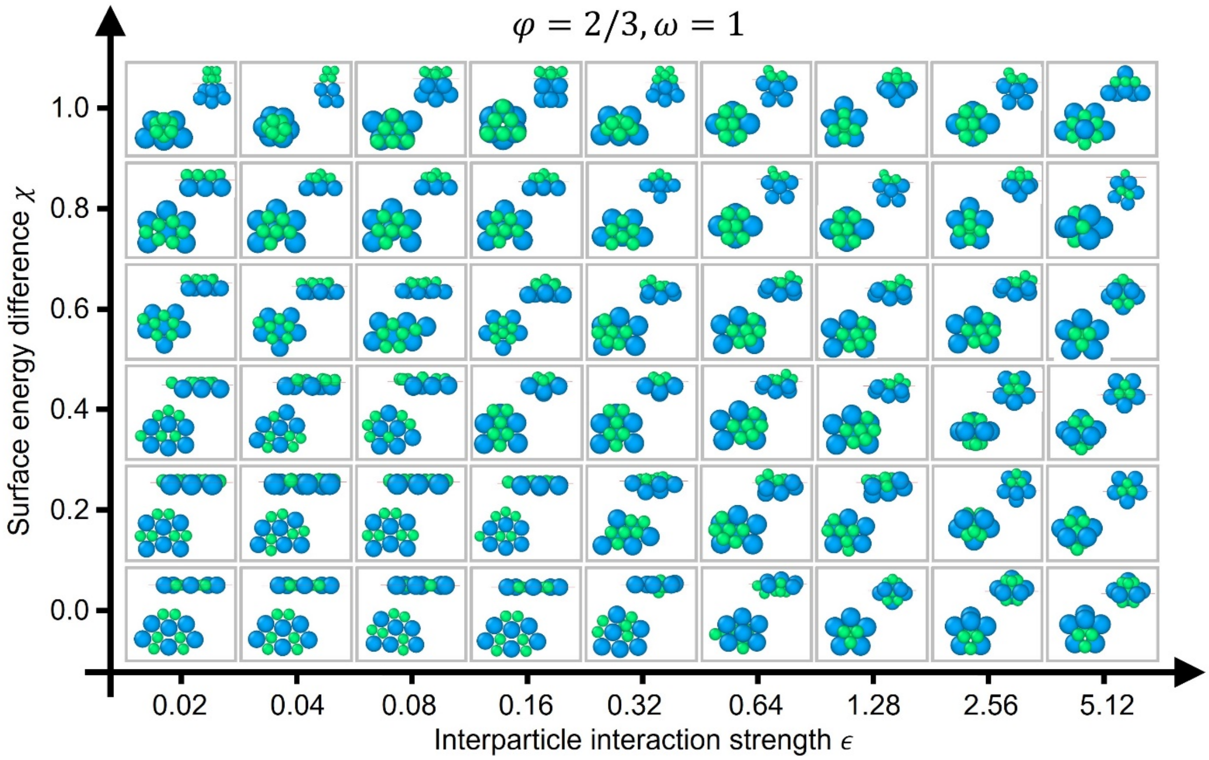

Supplementary Fig. 10 Structural phase diagram of 6:6 NPs (top and side views) at $\varphi = 2/3$ and $\omega = 1$ with respect to interparticle interaction strength parameter ϵ and relative miscibility parameter χ.

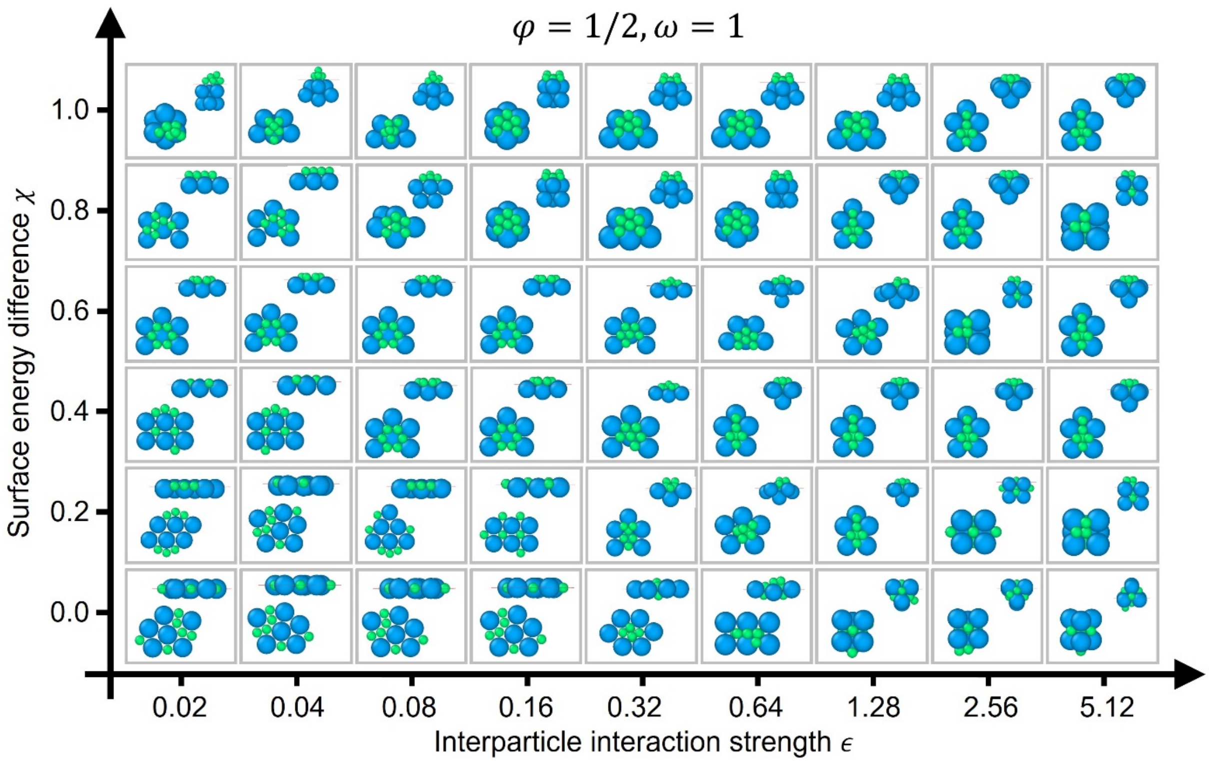

Supplementary Fig. 11 Structural phase diagram of 6:6 NPs (top and side views) at $\varphi = 1/2$ and $\omega = 1$ with respect to interparticle interaction strength parameter ϵ and relative miscibility parameter χ.

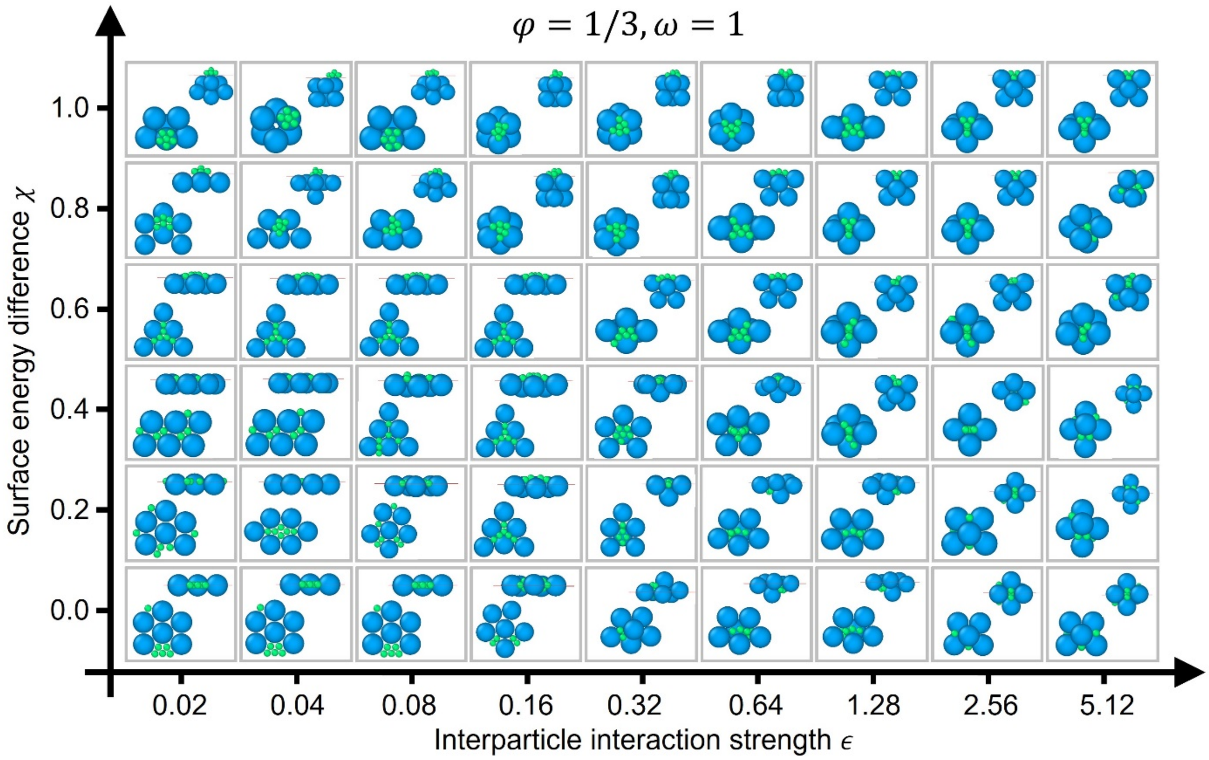

Supplementary Fig. 12 Structural phase diagram of 6:6 NPs (top and side views) at $\varphi = 1/3$ and $\omega = 1$ with respect to interparticle interaction strength parameter ϵ and relative miscibility parameter χ.

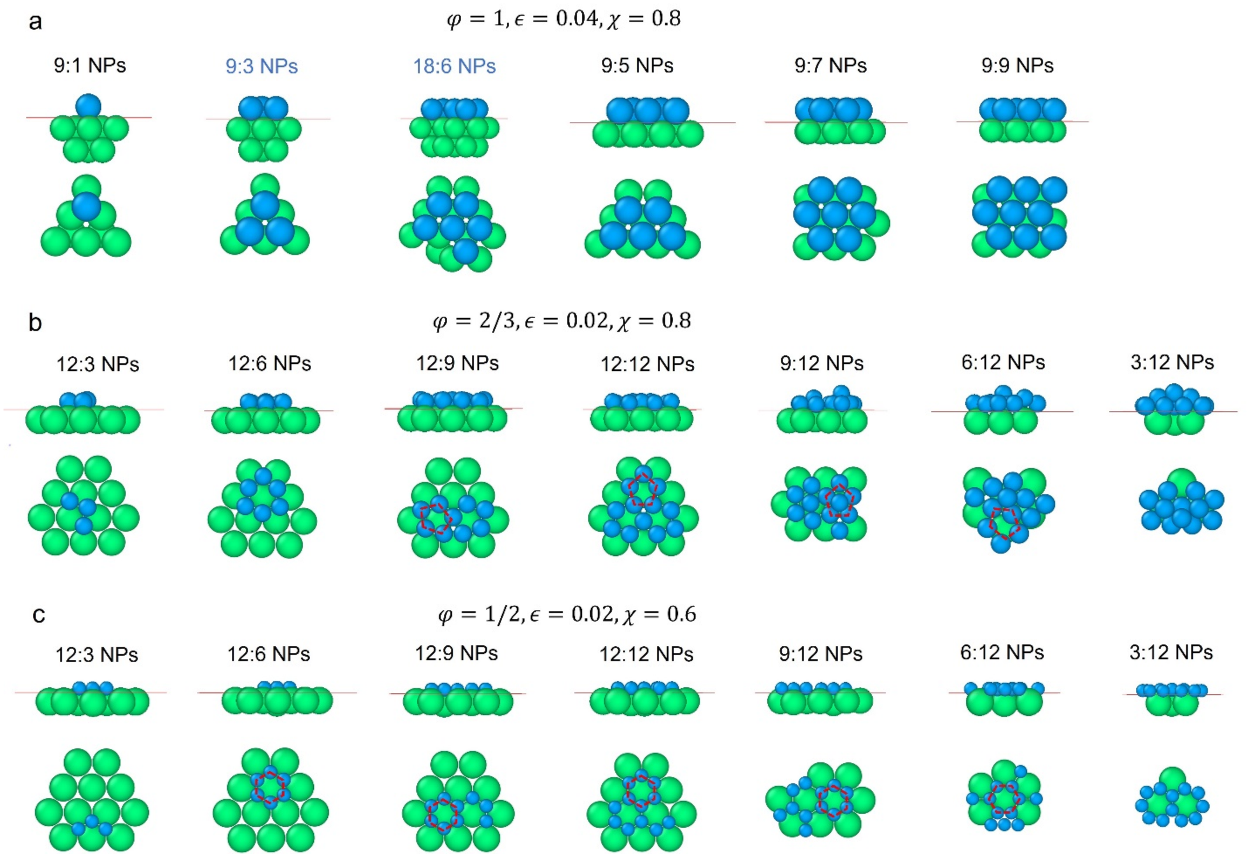

Supplementary Fig. 13 Impact of particle number ratio N_1:N_2 on the optimized structures. **a** Four additional ratios (note that 9:3 NPs is equivalent to 18:6 NPs) were explored for the hexagonally ordered BNSL in Fig. 2c obtained with 9:9 NPs. **b** Six additional ratios were explored for the BNSL with periodic tessellation of slender pentagons and triangles in Fig. 5b obtained with 12:12 NPs. **c** Six additional ratios were explored for the BNSL with periodic honeycomb lattice in Fig. 5d obtained with 12:12 NPs. These results show that the studied BNSLs are robust to large variations in particle number ratio, well beyond the stoichiometry stipulated by the lattice periodicity.

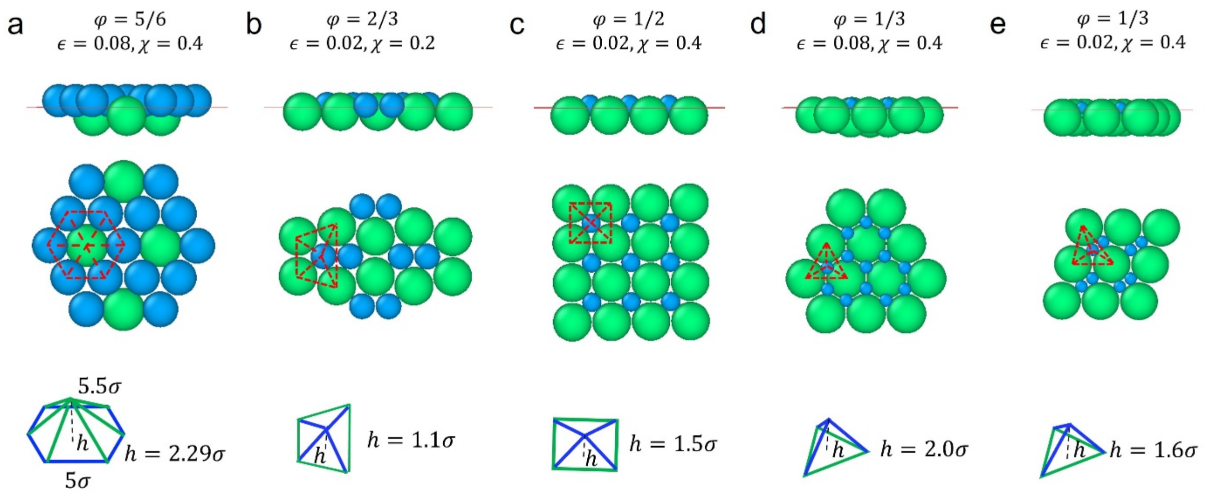

Supplementary Fig. 14 Monolayer BNSLs obtained at different particle size ratios. **a** AB_3-type BNSL with hexagonal order at $\varphi = 5/6$. **b** AB-type BNSL with stretched hexagonal order at $\varphi = 2/3$. **c** AB-type BNSL with square order at $\varphi = 1/2$. **d** AB_2-type BNSL with triangular order at $\varphi = 1/3$. **e** AB_2-type BNSL with rhombic order at $\varphi = 1/3$. The schematics at the bottom show the arrangement of one species of NPs around the other species. The vertical heights of the pyramids denoted by h provides the spacing between the two layers of NPs (in the direction normal to the interfacial plane).

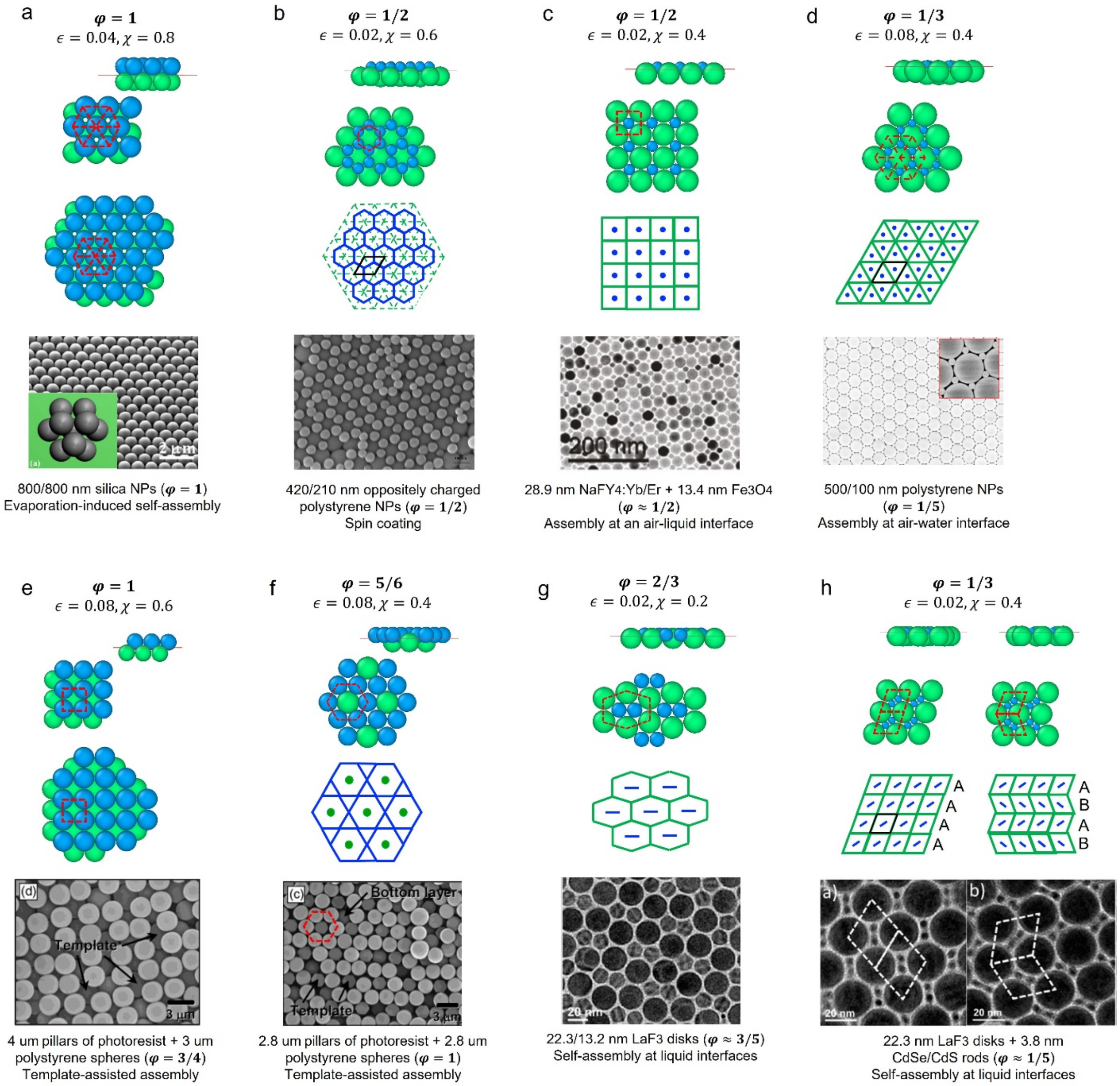

Supplementary Fig. 15 Comparison between our predictions and experiments. **a** AB-type hexagonally ordered bilayer. Experimental image is reprinted with permission from Ref. 2. Copyright 2012 IOP Publishing. Similar structures were also reported in Ref. 4. **b** AB_2-type honeycomb lattice. Experimental image is reprinted from Ref. 3, Copyright 2009, with permission from Elsevier. Similar structures were also reported in Refs. 4 and 5. **c** AB-type intertwined square lattices. Experimental image is reprinted with permission from Ref. 6. Copyright 2011 American Chemical Society. Similar structures were also reported in Refs. 5 and 7. **d** AB_2-type triangularly ordered BNSL. Experimental image is reprinted with permission from Ref. 8. Copyright 2016 American Chemical Society. Similar structures were also reported in Refs. 3, 5 and 6. **e**-**f** AB-type square bilayer (**e**) and AB_3-type Kagome lattice (**f**). Experimental images are reprinted with permission from Ref. 4. Copyright 2004 American Chemical Society. **g-h** Strained honeycomb (**g**) and rhombic (**h**) BNSLs. Experimental images are reprinted with permission from Ref. 9. Copyright 2015 American Chemical Society.

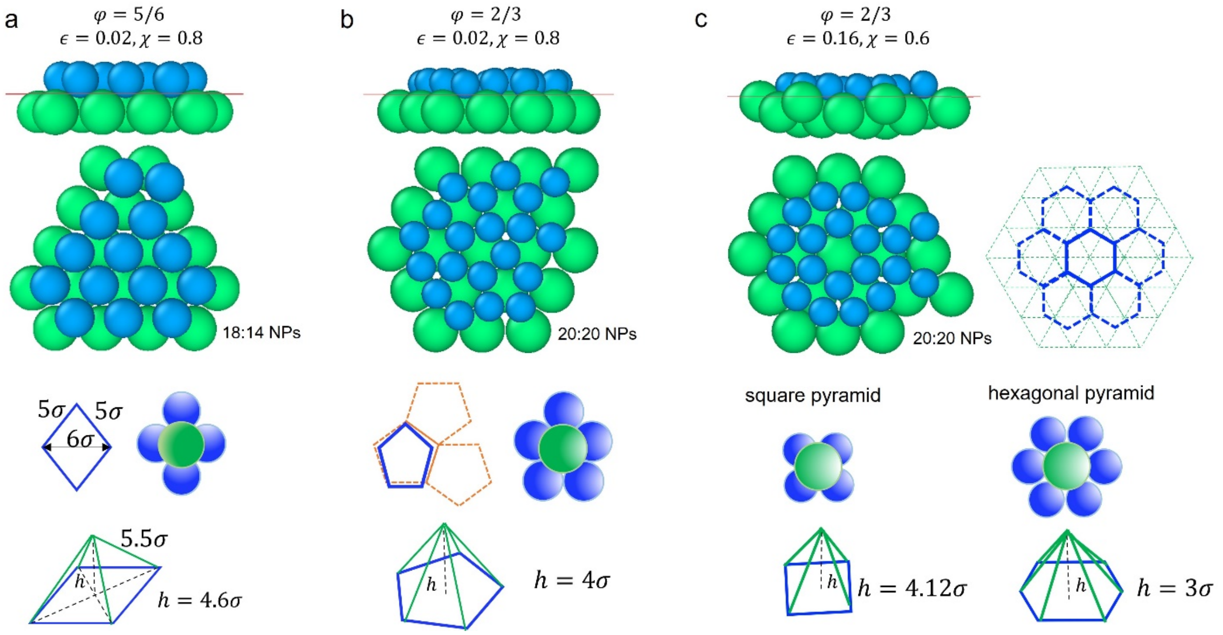

Supplementary Fig. 16 Hybrid bilayer BNSLs obtained at different particle size ratios showing how small NPs form unique quasi-periodic and periodic layers atop a hexagonally ordered sheet of large NPs. **a** AB-type BNSL with a quasi-periodic rhombic ordering of small NPs obtained at $\varphi = 5/6$. **b** A_3B_5-type BNSL with a periodic pentagonal-triangular arrangement of small NPs at $\varphi = 2/3$. Regular pentagons are unable to form a periodic tessellation, as schematically shown by the three pentagons in dashed brown lines. **c** A_4B_6-type BNSL made of periodic hexagonal, square, and triangular motifs at $\varphi = 2/3$. That hexagons on their own are unable to form a periodic arrangement on the hexagonally arranged lattice is schematically shown by blue and green dashed lines. In all cases, the NP numbers were deliberately chosen to be incompatible with the number of NPs in a unit cell to demonstrate robustness of the observed structures. The schematics at the bottom show the arrangement of one species of NPs atop the other species. The vertical heights of the pyramids, denoted by h, provide the spacing between the two layers of NPs.

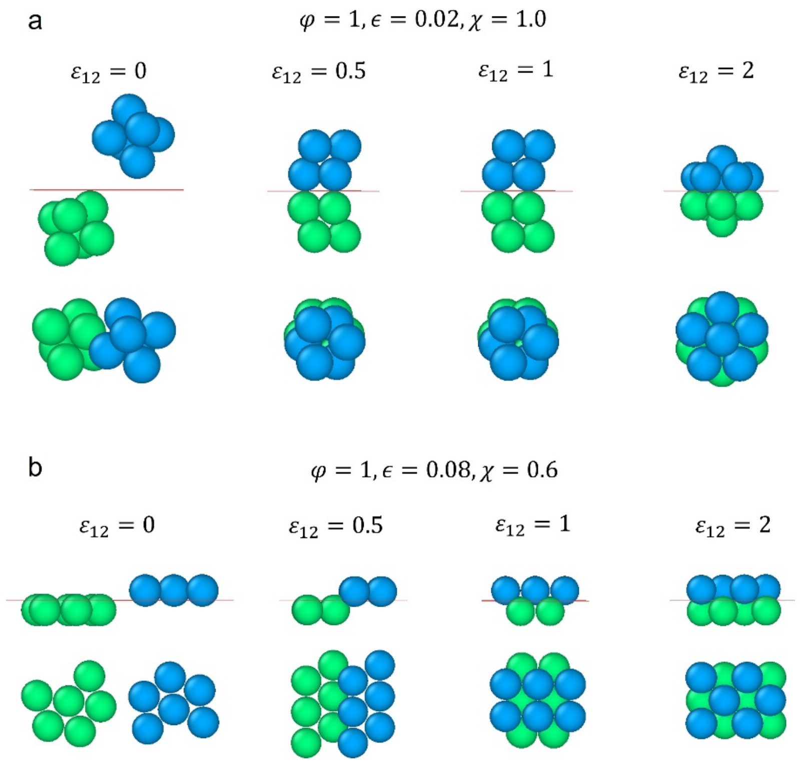

Supplementary Fig. 17 Effect of the inter-species interaction strength parameter ε_{12} on assembly of equal-sized NPs ($\varepsilon_{11} = \varepsilon_{22}$) with parameters $\epsilon = 0.02,\ \chi = 1$ (**a**) and $\epsilon = 0.08,\ \chi = 0.6$ (**b**). For $\varepsilon_{12} = \sqrt{\varepsilon_{11}\varepsilon_{22}} = 1$, assumed throughout this work, the NPs formed a quasi-globular cluster at $\epsilon = 0.02,\ \chi = 1$ and a square-ordered bilayer at $\epsilon = 0.08,\ \chi = 0.6$ (reproduced from Fig. 2a). However, when the attraction between NP 1 and NP 2 is entire removed ($\varepsilon_{12} = 0$), the two NP species form separated clusters at $\epsilon = 0.02,\ \chi = 1$ and separated hexagonal monolayers at $\epsilon = 0.08,\ \chi = 0.6$ in their respective fluid layers. When a weak attraction is introduced ($\varepsilon_{12} = 0.5$), NP 1 and NP 2 still form the same two clusters at $\epsilon = 0.02,\ \chi = 1$, but the two now connect with each other across the interface to gain the interactions between NP 1 and NP 2. Similarly, NP 1 and NP 2 still form individual hexagonal monolayers at $\epsilon = 0.08,\ \chi = 0.6$ but the two layers connect through their edges across the interface, where NPs can gain a few interactions between NP 1 and NP 2 without sacrificing their interfacial energy. When the attraction is made stronger ($\varepsilon_{12} = 2$), NP 1 and NP 2 form a single compact globular cluster at $\epsilon = 0.02,\ \chi = 1$ distinct from the original structure as the new structure attempts to maximize the number of contacts across the two species. Similarly, the two species form a single square bilayer when $\epsilon = 0.08,\ \chi = 0.6$, but distinct from the original bilayer in terms of NP-NP separation distance within each layer to again gain more interactions between NP 1 and NP 2.

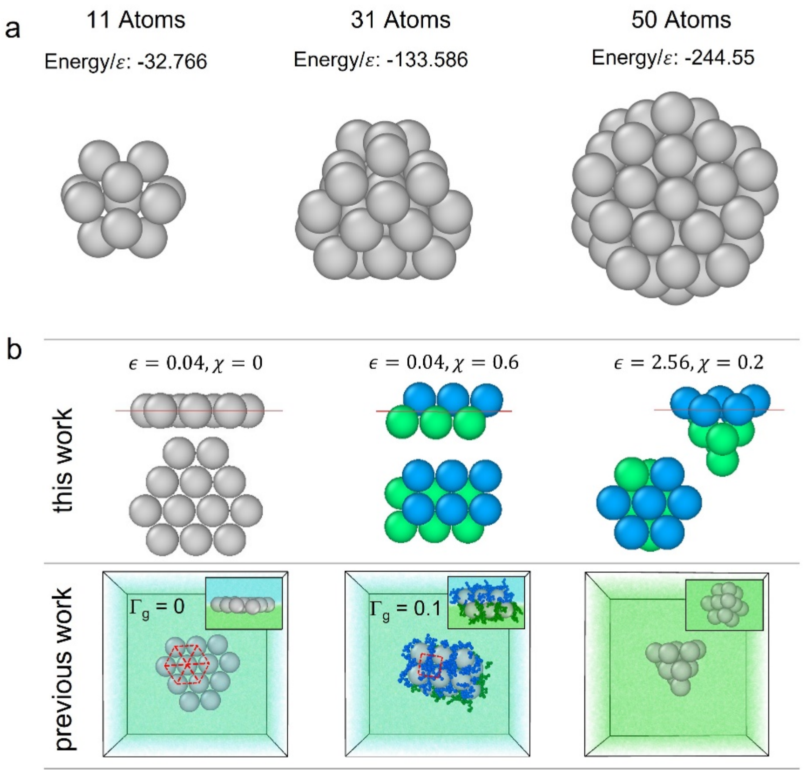

Supplementary Fig. 18 Validation of our optimization algorithm. **a** Ground-state structures of Lennard-Jones atom clusters of 11, 31, and 50 atoms and their corresponding energies obtained by our algorithm. **b** Comparison of interface-trapped structures obtained by our algorithm (for 12 NPs) at the specified conditions (top; reproduced from Fig. 2a) against those obtained from CGMD simulations (bottom) of bare and polymer-grafted NPs at a polymer-polymer interface or in bulk[1]. Left: hexagonally arranged monolayer; Middle: square bilayer; Right: globular cluster.

Supplementary References

1. Tang, T. Y., Zhou, Y. & Arya, G. Interfacial assembly of tunable anisotropic nanoparticle architectures. *ACS Nano* **13**, 4111–4123 (2019).

2. Sun, M. *et al.* Self-assembly nanoparticle based tripetaloid structure arrays as surface-enhanced Raman scattering substrates. *Nanotechnology* **23**, (2012).

3. Sharma, V., Yan, Q., Wong, C. C., Carter, W. C. & Chiang, Y.-M. Controlled and rapid ordering of oppositely charged colloidal particles. *J. Colloid Interface Sci.* **333**, 230–236 (2009).

4. Schaak, R. E., Cable, R. E., Leonard, B. M. & Norris, B. C. Colloidal crystal microarrays and two-dimensional superstructures: A versatile approach for patterned surface assembly. *Langmuir* **20**, 7293–7297 (2004).

5. Wang, D. & Möhwald, H. Rapid fabrication of binary colloidal crystals by stepwise spin-coating. *Adv. Mater.* **16**, 244–247 (2004).

6. Dong, A., Ye, X., Chen, J. & Murray, C. B. Two-dimensional binary and ternary nanocrystal superlattices: The case of monolayers and bilayers. *Nano Lett.* **11**, 1804–1809 (2011).

7. Ye, X. *et al.* Structural diversity in binary superlattices self-assembled from polymer-grafted nanocrystals. *Nat. Commun.* **6**, 1–10 (2015).

8. Lotito, V. & Zambelli, T. Self-Assembly of Single-Sized and Binary Colloidal Particles at Air/Water Interface by Surface Confinement and Water Discharge. *Langmuir* **32**, 9582–9590 (2016).

9. Paik, T., Diroll, B. T., Kagan, C. R. & Murray, C. B. Binary and Ternary Superlattices Self-Assembled from Colloidal Nanodisks and Nanorods. *J. Am. Chem. Soc.* **137**, 6662–6669 (2015).
